# Manganese Oxide Nanoparticles for MRI-Based Multimodal Imaging and Theranostics

**DOI:** 10.3390/molecules29235591

**Published:** 2024-11-26

**Authors:** Carlos F. G. C. Geraldes

**Affiliations:** 1 Department of Life Sciences and Coimbra Chemistry Center-Institute of Molecular Sciences (CQC-IMS), Faculty of Science and Technology, University of Coimbra, 3004-531 Coimbra, Portugal; geraldes@ci.uc.pt; Tel.: +351-967661211; 2 CIBIT—Coimbra Institute for Biomedical Imaging and Translational Research, University of Coimbra, 3004-531 Coimbra, Portugal

**Keywords:** contrast agents, functionalized nanoparticles, manganese oxides, magnetic resonance imaging, multimodal imaging, theranostics

## Abstract

Manganese-based MRI contrast agents have recently attracted much attention as an alternative to Gd-based compounds. Various nanostructures have been proposed for potential applications in in vivo diagnostics and theranostics. This review is focused on the discussion of different types of Mn oxide-based nanoparticles (Mn_x_O_y_ NPs) obtained at the +2, +3 and +4 oxidation states for MRI, multimodal imaging or theranostic applications. These NPs show favorable magnetic properties, good biocompatibility, and an improved toxicity profile relative to Gd(III)-based nanosystems, showing that the Mn paramagnetic ions offer advantages for the next generation of nanoscale MRI and theranostic contrast agents. Their potential for enhancing relaxivity and MRI contrast effects is illustrated through discussion of selected examples published in the past decade.

## 1. Introduction

The recent advances in the synthesis and physical-chemical characterization of complex functionalized nanoparticles (NPs), as well as in the understanding of how these properties depend on their size, shape and surface modifications, have enabled their application in biomedicine, such as diagnostics and therapeutics. Their main advantages relative to small molecules are: (a) incorporation or surface conjugation with multiple types of imaging or therapeutic agents, forming versatile nanoplatforms with a suitable design for diagnostic or theranostic applications; (b) surface modification with hydrophilic polymers such as polyethylene glycol (PEG), enhancing their stealth properties and prolonging their circulation time; (c) inclusion of targeting molecules to guide the nanoprobes to specific disease sites for drug delivery and imaging. These characteristics make multifunctional NPs highly promising as innovative diagnostic and therapeutic probes in the field of biomedicine [1,2,3,4,5,6,7,8,9,10].

Among the many and complementary imaging techniques available for preclinical studies, such as magnetic resonance imaging (MRI), optical imaging (OI), computed tomography (CT), positron emission tomography (PET), single-photon emission computed tomography (SPECT) and ultrasound (US), MRI has an important role as a non-invasive diagnostic technique with high spatial resolution (mm scale), exceptional intrinsic contrast, no limitation in tissue penetration and non-use of ionizing radiation in image acquisition [11,12]. The central role of MRI in clinical diagnostics was favored by the development of effective contrast agents (CAs), which are routinely used in diagnosis to improve image contrast and reduce the acquisition time [13,14]. The clinically used MRI CAs consist of hydrophilic, low molecular weight paramagnetic complexes consisting of Gd^3+^ ions bound to octadentate open chain and macrocyclic polyaminocarboxylic ligands, and with a water molecule in the inner coordination sphere of the metal ion, in fast exchange with the bulk water [15]. The paramagnetic effect from the Gd^3+^ ion is transferred to the surrounding solvent molecules by this exchange process, resulting in shorter values of the nuclear magnetic relaxation times (T_1_ and T_2_) of the water protons in the vicinity of the CAs, leading to a variation in the MRI signal intensity and generating an increase in image contrast [16].

However, the limited sensitivity and low temporal resolution of MRI have hindered its molecular imaging clinical application. Thus, NP-based MRI CAs have been explored to significantly enhance the sensitivity of MRI and reduce the required injected dose for detectable image contrast. MRI can be combined with other modalities with complementary advantages, such as the high sensitivity provided by PET, in a single nanoplatform, to obtain better functional and anatomical imaging information.

Gd(III)-based nanosystems are the most extensively studied for T_1_-weighted MRI, consisting of a very large number of Gd(III) complexes (103–105 per particle) incorporated into or conjugated to nanocarriers, or Gd^3+^ ions being an integral part of the inorganic nanostructure [17,18,19,20,21,22]. The incorporation of a large number of active paramagnetic centers within the NPs enhances their efficiency (r_1_ relaxivity), particularly in the magnetic field range of 0.5–2 T. This improvement results from the increased molecular volume of the nanoprobe, which leads to a slowing down of its global tumbling motion, with rate 1/τ_R_ [19], where τ_R_ is the correlation time for the rotational tumbling of the NP. The preparation, properties, and applications of these nanosized Gd-based NPs (organic-based, inorganic and hybrid) as MRI CAs have been extensively investigated and comprehensively reviewed [17,18,19,20,21,22].

However, while Gd(III) chelates used in clinical practice as MRI CAs (gadolinium-based contrast agents, GBCAs) are generally considered safe and well-tolerated by patients, there have been two clinical concerns in recent years. Their use in patients with severe chronic kidney disease (CKD) was demonstrated to cause nephrogenic systemic fibrosis (NSF), a rare progressive disease involving fibrosis of the skin and connective tissues, leading to skin thickening and joint contractures, eventually extending to internal organs such as the liver, lungs, heart, and muscles, and potentially causing severe disability or death [23]. It has been related to the in vivo displacement of Gd^3+^ from the chelating agents by transmetalation reactions which release the highly toxic free Gd^3+^ ions, particularly in the less kinetically stable linear GBCAs [24]. Another issue has been the observation of a concentration-dependent deposition and retention of gadolinium in the human brain in trace amounts upon repeated use of GBCAs, which were seen as high signal intensities in the globus pallidus and dentate nucleus on unenhanced T_1_-weighted images. Postmortem human or animal studies have validated gadolinium deposition in trace amounts in these areas [25]. The macrocyclic GBCAs were far less retained in the brain than the linear ones, and the entire detected gadolinium was intact soluble GBCA, while the linear ones showed both soluble and insoluble gadolinium-containing species, with a dominance of the insoluble species, showing the importance of in vivo kinetic stability of these agents [26]. These findings raised new concerns on the safety of GBCAs, in particular the linear ones, that have prompted research efforts to explore safer alternatives [27]. Therefore, although the studies of Gd-NP-based MRI CAs are still in the preclinical phase, a shift in research is occurring towards exploring the use of other paramagnetic ions, such as the transition metal ions Mn(II/III/IV) and Fe(III), both as small complexes and nanoprobes [28,29,30,31].

Both Fe(III) and Mn(II) are essential elements found in all known living organisms and are involved in many metabolic processes. However, exposure to high levels of these metal ions can lead to adverse health effects. When using Fe(III)-based systems as MRI CAs, the presence of the free metal ions should be avoided, in particular Fe(II), which can form toxic free radicals, such as superoxide and hydroxyl reactive oxygen species through the Fenton reaction, and its concentration in body tissues must be tightly regulated because in excessive amounts, it can lead to tissue damage [32]. Fe(III)-based MRI CAs have been developed mainly as superparamagnetic iron oxide nanoparticles (SPIONs), which have primarily been applied as negative CAs for T_2_w MRI applications [33]. However, there are some limitations in their use due to the blooming artifacts they can produce due to regions of high magnetic susceptibility differences, which appear as signal spreading or blooming beyond the actual boundaries of those regions.

Whereas high levels of Fe have been associated with neural diseases such as Alzheimer’s, Parkinson’s and Huntington’s, excess Mn^2+^ in the body is correlated to manganism, attention deficit hyperactivity disorder, depression, and hepatic encephalopathy [34,35,36]. However, as free Gd^3+^ is much more toxic than Mn^2+^ and Fe^2+^, as shown by the LD_50_ (rat, oral) values of GdCl_3_ (0.1 mM kg^−1^), MnCl_2_ (71 mM kg^−1^) and FeEDTA (3.7 mM kg^−1^), manganese oxide NPs offer potential solutions to the limitations of Gd^3+^ oxide NPs, such as lower toxicity related to cation release and possible improved biocompatibility with appropriate surface protection.

In this article, we review recent studies on manganese oxide-based nanoparticles as MRI CAs, in particular their surface modification or functionalization to improve their properties, such as effectiveness as relaxation probes, dispersibility, targeting capabilities and multifunctionality, including multimodal imaging and drug delivery capabilities, while maintaining low toxicity.

## 2. Basic Properties of Manganese Ions

Manganese is a transition metal with a [Ar]3d^5^4s^2^ electronic configuration with possible oxidation states ranging from 0 to +7. The paramagnetic properties of the Mn ions are determined by the number of unpaired electrons distributed in its five d orbitals. Thus, the Mn oxidation states II, III and IV are the relevant ones for the design of MRI CAs. In its divalent, high spin 3d^5^ state, Mn(II), is a very efficient paramagnetic relaxation agent with a large electronic spin quantum number, S = 5/2, high effective magnetic moment (μ_eff_ = 5.92 μB/atom), long electronic relaxation time (0.1–1 ns range), and quite fast exchange rates of the coordinated water molecules (Table 1). Typical donor atoms for Mn^2+^ with coordination number 6 (ionic radius = 0.83 Å) are carboxylate oxygen and amino nitrogen atoms. Some Mn(II) complexes also have reasonable chemical stability around physiological pH. Despite these favorable properties as MRI CAs in alternative to Gd(III) chelates [28,37], only one Mn(II)-based CA has been approved for clinical use (mangafodipir trisodium, [Na_3_[Mn(H_3_DPDP)], TESLASCAN^®^) [38]. This results in part from the difficulty in achieving high thermodynamic stability and kinetic inertness in Mn(II) chelates due to the smaller charge and the absence of crystal field stabilization energy in high spin Mn(II) ions. Exceptions to this are the Mn(II) complexes of PyC3A [39] and of 2,4-pyridyl-disubstituted bispidine ligands [40].

The high spin Mn(III) ion, with 3d^4^ configuration and S = 2, has lower effective magnetic moment (μ_eff_ = 4.9 μB/atom) and Mn(III) compounds are less efficient relaxation agents due to the shorter electronic relaxation times (ca. 10 ps), although the water exchange is also fast. The harder and smaller Mn^3+^ cation (0.64 Å) is not sufficiently stable in aqueous solutions unless coordinated with suitable multidentate ligands, preferably with strongly electron-releasing donor atoms such as in phenolate or catecholate groups, or in macrocyclic ligands such as porphyrins and phthalocyanines, where Mn^3+^ is octa-coordinated by four equatorial nitrogen atoms of the ligand and two water molecules in an axial position (q = 2), leading to high r_1_ relaxivity values [41].

Mn(IV) has the 3d^3^ configuration with three unpaired electrons and lower paramagnetism (S = 3/2, μ_eff_ = 3.87 μB/atom). It usually occurs as MnO_2_-like NPs, where the contrast enhancement results from the release of free Mn^2+^ after dissolution of the particle triggered by endogenous stimuli [42].

Moreover, Mn easily forms multiple magnetic ions and complexes reacting, for example, with oxygen to form Mn oxides such as MnO, Mn_3_O_4_, Mn_2_O_3_ and MnO_2_, in which Mn shows +2, mixed +2/+3, +3, and +4 oxidation states, respectively.

## 3. Manganese Oxide-Based Nanoparticles as MRI Contrast Agents

Considering Mn(II)-based nanosystems, there are three main classes of NPs that have been investigated in the literature: different types of Mn(II)-chelates anchored on or embedded into soft and/or hard nanostructures, or other inorganic nanoparticles (i.e., KMnF_3_, MnWO_4_, etc.), which are not the subject of this review; T_1_ Mn(II)-oxide (MnO)-based NPs, which are discussed in detail; and Mn(II) ferrite (MnFe_2_O_4_) nanoparticles, which are only briefly discussed here.

### 3.1. MnO Nanoparticles

Several reviews have covered the topics of preparation, characterization, MRI, and toxicological properties of MnO NPs [9,42,43,44], as well as the chemistry and MRI capabilities of responsive MnO-based contrast agents [42]. However, some MnO-based NPs frequently exhibit long-term Mn^2+^ release, resulting in the accumulation of free Mn^2+^ in tissues and associated toxic effects, as demonstrated by several toxicological studies [45,46,47,48]. Here, some recent representative examples of applications of such MnO-based NPs as targeted, responsive, uni- and multimodal, and theranostic MRI CAs are described, and the characteristics of some of them are summarized in Table 2.

#### 3.1.1. MnO-Based NPs as MRI CAs

In the case of MnO NPs, the Mn^2+^ ions exposed on the surface are the only ones in magnetic contact with bulk water molecules, thus dominating the contrast enhancement, and their number increases with the surface-to-volume ratio of the NPs. Thus, in recent years, much work has focused on engineering the surface of MnO NPs to increase T_1_ relaxivities (r_1_) and improve the accuracy of diagnosis [29,42]. However, their relatively low r_1_ values imply the need for high dosage MnO NPs to clearly detect a lesion, increasing the danger of unfavorable consequences and limiting the application of MnO NPs in clinical diagnosis [49,50].

MnO NPs of sub-10 nm size may display a crystalline surface, highly related to the morphology, and surface coating ligands that can be employed to enhance the water content surrounding the NPs. As a result, since nanostructures with anisotropic morphology may exhibit significantly higher surface-to-volume ratio than their spherical counterparts, MnO NPs of different shapes and sizes have been synthesized, ranging from spheres [51] to cubes [52] and many other non-spherical morphologies [53], with sizes typically lower than 10 nm to obtain a low T_1_/T_2_ relaxivity ratio (r_2_/r_1_) and thus T_1_ contrast ability. However, non-spherical nanoparticles have often shown rather undesirably high r_2_/r_1_ values; hence, a correct analysis of surface-water interactions is needed to elucidate the relationship between crystal surface and T_1_ relaxivity and optimize the contrast enhancement ability of the NPs [54]. Surface coating is also typically needed to improve the in vivo pharmacokinetics of NPs; thus, MnO NPs have been functionalized with polyethylene glycol (PEG) [55,56], functional polymers, e.g., PMA (poly-(isobutylene-alt-maleic anhydride) or PLGA (poly(lactic-co-glycolic acid) [57], biocompatible lipid liquid crystalline NPs (LLCNPs) [58], coated with silica [59], or modified by phospholipids [60].

PEG-coated MnO NPs with a hydrodynamic diameter of ca. 15 nm were conjugated to the AS1411 aptamer, which allowed targeting 786–0 renal cancer tumor cells and prolonging the accumulation time of the probe in tumor cells. At 3.0 T, this nanoprobe had an r_1_ value of 12.9 mM^−1^ s^−1^ and an r_2_/r_1_ ratio of 4.66 [55]. Fortin et al. showed that PEGylated bis-phosphonate dendrons (PDn) grafted on the surface of MnO NPs (MnO@PDn) could be used as T_1_ vascular contrast agents due to a fast excretion capacity of the NPs [56]. The same group described Mn-silica nanohybrid materials by confining MnO into the porous network of MCM-48 silica nanoparticles (MnM48SNs) [61]. In mild acidic media, Mn–M48SNs did not show significant evidence of Mn^2+^ release, although leaching of Mn^2+^ was observed in acetate buffer at pH 5. Longitudinal relaxivity values of r_1_ = 8.4 mM^−1^ s^−1^ were reported (1.5 T and 310 K), with a low r_2_/r_1_ = 2. Finally, leukemia cells were labeled with Mn–M48SNs and the MRI contrast enhancement provided was quantified at 1 T.

Targeting agents were also conjugated to the surface of MnO NPs for cancer imaging applications: for example, Lee et al. reported folic acid-conjugated, polyacrylic acid (PAA)-coated MnO NPs with an average particle size of 2.7 nm [62]. While PAA provided good colloidal stability and low cellular cytotoxicity, folic acid gave cancer-targeting ability, confirmed by T_1_ contrast enhancement at the U87MG brain cancer site upon intravenous (i.v.) injection to mice tails. Further, the NPs displayed a quite high r_1_ value of 9.3 mM^−1^ s^−1^ (r_2_/r_1_ = 2.2) at 3.0 T. Long and co-workers synthesized PEG-coated MnO NPs modified with RGD peptide to target tumors via α_v_β_3_ integrin [63]. In this case, an r_1_ value of 12.1 mM^−1^ s^−1^ and an r_2_/r_1_ ratio close to 1 were measured at 4.7 T. They also investigated the effect of two different PEG chain lengths (600 and 5000 Da) in the performance of MnO NPs in vivo, showing that PEG 5000-modified NPs underwent higher tumor accumulation. Furthermore, Huang et al. also described MnO NPs PEGylated via Mn(II) chelation by catechol moieties and conjugated to cRGD as active tumor targeting group [64]. They reported a larger size of ca. 100 nm after coating and RGD conjugation, a slightly high r_2_/r_1_ ratio of ca. 6 (r_1_ = 10.2 mM^−1^ s^−1^ at 3.0 T and 310 K), and an efficient accumulation in tumor verified by an in vivo biodistribution study. Finally, Wei et al. reported ultrasmall MnO NPs coated with zwitterionic dopamine sulfonate (ZDS) (USMnO@ZDS) with a much better capability of MRI T_1_-weighted contrast enhancement (r_1_ = 15.6 mM^−1^ s^−1^ at 0.5 T) than conventional large-sized MnO NPs, with improved biocompatibility and prevented opsonization by serum albumins. They are capable of passing through the blood−brain barrier (BBB), showing promise for accurate diagnosis of brain diseases [65].

#### 3.1.2. MnO-Based NPs as Multimodal MRI CAs

One way to design bimodal MRI CAs is through dual-mode T_1_–T_2_ MRI contrast agents (DMCAs), which are able to induce MRI images of the same region with positive or negative contrast using the same MRI scanner and T_1_-weighted (T_1w_) and T_2_-weighted (T_2w_) pulse sequences, significantly improving their efficiency and diagnostic accuracy. For optimal dual contrast, they should have both high r_1_ and r_2_ values, with r_2_/r_1_ in the 2–10 range. One of the approaches to design efficient DMCAs is to include both types of T_1_ and T_2_ contrast materials in the same nanoplatform by changing their relative distances and positions [66]. To avoid signal quenching, their distance should be greater than 20 nm and the T_1_ contrast material used should be located inside the T_2_ contrast material, as in this configuration their local magnetic field strengths are mutually reinforced [67].

**Figure 1:**
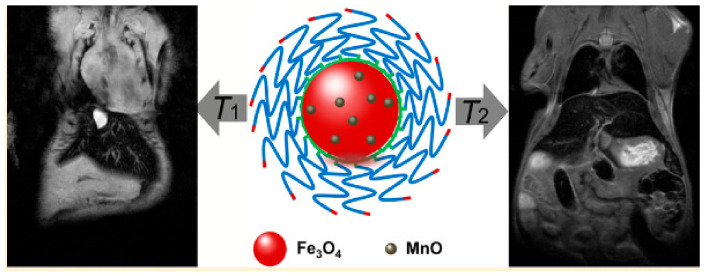
Center: structure of the MnO@Fe_3_O_4_-OH-PEG-PH NPs; Simultaneous in vivo T_1w_ MR image (left), and in vivo T_2w_ MR image (right) of a BALB/c mouse 1 h after injection in the tail vein of MnO@Fe_3_O_4_-OH-PEG-PH NPs (1.5 mg [Fe] kg^−1^). Reproduced with permission from Ref. [68]. Copyright (2019) by the American Chemical Society.

The different methods to prepare DMCAs by embedding MnO (paramagnetic T_1_ contrast material) into Fe_3_O_4_ (superparamagnetic T_2_ contrast material) have been extensively studied [36,58]. As a recent example, Lu et al. reported hydroxyl−PEG−phosphonic acid-stabilized spherical Fe_3_O_4_/MnO (MnO@Fe_3_O_4_-OH-PEG-PH) NPs, highly water dispersible due to the hydrophilic surface coating, and with 21 nm hydrodynamic size as obtained by dynamic light scattering (DLS) technique. Their values of r1= 22.8 mM^−1^ s^−1^ and r_2_ =209.6 mM^−1^ s^−1^ (vs [Fe + Mn]) measured in in vitro phantoms at 0.5 T, gave an r_2_/r_1_ ratio of 9.2. Their efficiency as T_1_-T_2_ DMCAs was confirmed by the observed gradual brightening of the T_1w_ MRI images and the gradual darkening of the T_2w_ MRI images (7 T) of the liver of BALB/c mice upon i.v. injection of the NPs [68] (Figure 1).

The characteristics of MRI and the other main imaging modalities show that each one has intrinsic advantages and limitations, which sometimes makes it difficult to obtain accurate and reliable information at the pathological site. Spatial resolution is high for MRI and CT and lower for PET/SPECT and OI. However, sensitivity is extremely high in PET/SPECT, high in OI, medium in US and MRI and low in CT. Temporal resolution is lowest for MRI. Penetration depth is unlimited for MRI, CT and PET/SPECT, medium for US, but is limited to a few centimeters in OI. The image contrast of soft tissues is better for MRI, PET, SPECT and PAI than for CT and US. Other important technical properties are the possibility of multicolor imaging in OI, quantitative imaging in PET and whole-body imaging in PET/SPECT, MRI and CT [10]. Despite the advantages of MRI, namely superb spatial resolution, good image contrast of soft tissues and unrestricted tissue penetration, some of its limitations, such as low time resolution and low sensitivity, affect their clinical application. Therefore, multimodal MRI CAs can be designed by integrating MRI with other modalities with complementary advantages in a single nanoplatform, such as the high sensitivity provided by PET and optical imaging (OI), despite the low penetration depth of the latter, which can lead to improved anatomical and functional imaging information.

In the examples discussed in this section and the next one, summarized in Table 2, it is exemplified how manganese oxide-based nanoplatforms with multimodal and therapeutic capabilities can improve diagnostic accuracy and treatment efficacy, potentially enhancing sensitivity and reducing false positives.

MnO-based nanoplatforms function efficiently as bimodal MRI CAs by including a probe active at another imaging modality, with complementary properties to MRI, to overcome the intrinsic limitations of the MRI modality. An example is the integration of the MRI and OI modalities to obtain high sensitivity and resolution simultaneously, becoming more efficient for accurate MRI detection of tumors. Chen et al. prepared the MRI/near-infrared imaging bimodal nanoprobe (MnO-PEG-Cy5.5), by conjugating the near-infrared (NIR) dye Cy5.5 to the MnO surface using PEG-Cy5.5. This nanoplatform, of 20.9 nm size, had in vitro r_1_= 5.73 mM^−1^ s^−1^ and r_2_ =40.15 mM^−1^ s^−1^ (vs [Fe + Mn]) at 0.5 T, and r_2_/r_1_ of 7.0. In vivo T_1w_ MRI (7 T) positive contrast enhancement by the injected MnO-PEG-Cy5.5 nanoprobe was demonstrated in the tumor region of a glioma bearing nude mouse, which was confirmed by whole-body NIRF imaging [69].

As an example of the optimization of MRI-based multimodal probes, Zhang et al. used a novel strategy, combining structure design and composition design to maximize the imaging ability of each building block, and a sequential self-reduction reaction (SSR) synthetic approach to develop a US/MRI/CT trimodal nanoprobe. It consists of hollow mesoporous silica nanoparticles (HMSNs) of around 400 nm in diameter with a large Au NP and some smaller Au nanocrystals in the hollow cavity and mesoporous shell of HMSNs, respectively, with small MnO NPs uniformly distributed in the mesoporous shell, which is functionalized with positively charged polyethylenimine (Au@HMSN@Au-MnO-PEI). Upon in vivo evaluations using VX2 tumor–bearing rabbits, this biocompatible trimodal probe demonstrated excellent performance in intensifying CT, T_1w_-MRI and US tumor contrast, showing potential in guiding designs of other materials-based multimodal imaging probes [70].

#### 3.1.3. MnO-Based NPs as MRI-Guided Theranostic Agents

MnO NPs can be modified by loading therapeutic drugs for MRI-guided tumor targeted drug delivery with increased accuracy and reduced toxic side effects, as well as adapted for multimodal imaging. A recent example used hollow porous MnO (HPMO) NPs, obtained by acid or basic etching, which exhibit a high surface-to-volume ratio compared to their solid equivalents and therefore increased relaxivities. Wei et al. reported small-sized octapod-shaped HPMO NPs modified with zwitterionic dopamine sulfonate (ZDS) that could serve as a versatile platform for efficiently loading of the organic dye and the chemotherapeutic (ChT) drug DOX. The MRI contrast enhancement was achieved by release of free Mn^2+^ ions in mild acidic conditions. The pH-induced T_1_ signal and the fluorescence recovery behavior of the cargo@HPMO allowed real-time monitoring of the release of cargoes in vitro in SMMC-7721 cells and in vivo in H22 tumor-bearing mice, especially in the acidic tumor microenvironment (TME) and lysosomes [71]. Another example is from Bae et al., who used hollow manganese oxide nanoparticles coated with PEI-DOPA and conjugated with the therapeutic monoclonal antibody, herceptin and with siRNA (HMON-PEI-HER/siRNA) to target mRNA degradation in T_1w_ MRI, as well as the efficient intracellular delivery of siRNA for cell-specific gene silencing [72]. Yang and colleagues designed an acidity-sensitive nanotheranostic agent (FePt@MnO)@DSPE-PEG5000-FA, FMDF NPs) for T_1_/T_2_ MRI and CT guided ferroptosis chemodynamic therapy (CDT) in a 4T1 mouse cancer model. The NPs could specifically target folic acid (FA) receptor-positive tumor cells and induce ferroptosis efficiently by rapidly releasing Fe^2+^ to catalyze the Fenton reaction of intracellular H_2_O_2_ into ROS. Due to the acidity of the TME, the release of Mn^2+^, Fe^3+^ and Pt^2+^ ions provided T_1_/T_2_ MRI and CT contrast in the tumor of 4T1 mice, as well as suppressing tumor growth [73]. Finally, Zhou et al. reported a multifunctional theranostic nanoplatform consisting of MnO NPs covered with the amphiphilic copolymer PSMA–PEG and the IR808 dye (MnO@PSMA-PEG-Ir808), for in vivo photothermal therapy (PTT). These NPs (40 nm hydrodynamic size) were recognized and assimilated by a tumor cell membrane protein using IR808 as a tumor-targeting ligand, and located in the mitochondria. The MnO reacted with the mitochondrial endogenous H_2_O_2_ in the acidic environment (pH~5.5) of the TME to produce free Mn^2+^ ions, O_2_ and free radicals (OH,⋅O_2_^−^), relieving tumor hypoxia and inducing mitochondrial damage and cell apoptosis, respectively. Moreover, under NIR 808 nm laser irradiation, the IR808 unit of the NPs produced highly toxic singlet oxygen ^(1^O_2_) and generated hyperthermia to kill the cancer cells. The tumors of MCF-7 nude mice, treated with the NPs by injection in the tail vein, were completely attenuated under 808nm near-infrared (NIR) light, as observed by T_1w_-MRI, photoacoustic imaging (PAI) and optical imaging (OI) [74].

**Table 2:** Overview of fundamental characteristics of some of the examples of MnO-based NPs as MRI (and multimodal/theranostic) CAs covered in this section.

NPs Components	Imaging Modalities	Relaxivity (mM^−1^ s^−1^), 298 K ^a^, B_o_ (T)	Therapeutic Modalities	Cell/Animal Model	Reference
MnO-PEG-AS1411 aptamer	Targeted T_1_ MRI	r_1_ = 12.9, r_2_/r_1_ = 4.66 (3 T) ^b^	-	786-0 cells on BALB/c mice	[55]
MnO@PDn	Vascular T_1_ MRI	r_1_ = 4.4, r_2_/r_1_ = 8.6; (1.41/T, 310 K)	-	Balb/c mice	[56]
MnM48SNs	T_1 *_ MRI	r_1_ = 8.4, r_2_/r_1_ = 2 (1.5 T, 310 K)	-	In vitro P388 leukemia cells	[61]
MnO-PAA-FA	Targeted T_1_ MRI	r_1_ = 9.3, r_2_/r_1_ = 2.2 (3 T) ^b^	-	U87MG brain tumor	[62]
MnO-PEG-RGD	Targeted T_1_ MRI	r_1_ = 12.1, r_2_/r_1_~1 (4.7 T) ^b^	-	M21 tumor cells on Balb/c mice	[63]
mPEG and cRGD-g-Pasp@MnO-	Targeted T_1_ MRI	r_1_ = 10.2, r_2_/r_1_ = 6 (3 T, 310 K)	-	A549 tumor-bearing mice	[64]
US MnO@ZDS	Targeted T_1_ MRI	r_1_ = 15.6, r_2_/r_1_ = 1.7 (0.7 T) ^b^	-	BALB/c mice	[65]
MnO@Fe3O4-OH-PEG-PH	T_1_-T_2_ MRI	r_1_ = 22.8, r_2_/r_1_ = 9.2 (0.5 T) ^b^	-	BALB/c mice	[68]
MnO-PEG-Cy5.5	NIRF/T_1_ MRI	r_1_ = 5.73, r_2_/r_1_ = 7.0 (7 T) ^b^	-	Glioma-bearing nude mice	[69]
Au@HMSN/Au_MnO-PEI	US/MRI/CT	r_1_ = 1.5, r_2_/r_1_ = 35.3 (3.0 T)	-	VX2 tumor–bearing rabbits	[70]
HPMO-ZDS NPs + DOX	MRI/OI	r_1_ = 0.8 (pH 7.4) → 8.3 (pH 5.4 (0.5 T)) (r_1_ of free Mn^2+^ ions) ^b,c^	ChT	SMMC-7721 cells H22 tumor-bearing mice	[71]
HMON-PEI-DOPA-HER/siRNA	T_1_-MRI	^b,c^	ChT	SK-BR-3 and MCF-7 cells	[72]
FePt@MnO)@DSPE-PEG5000-FA	T_1_-T_2_ MRI/CT	r_1_ = 4.16, r_2_/r_1_ = 1.2 (pH 7.4) → 6.43, 1.3 (pH 4.8 (3 T)) ^b^ (r_1_ of free Mn^2+^ ions)	CDT	Balb/c 4T1 tumor-bearing mice	[73]
MnO@PSMA-PEG-Ir808	MRI/NIR-OI/PAI	r_1_ increase due to release of free Mn^2+^ ions ^b,c^	PTT	MCF-7 tumor-bearing mice	[74]

^a^ Unless otherwise stated; ^b^ Temperature not indicated; ^c^ r_i_ not determined.

#### 3.1.4. Mn(II) Ferrite Nanoparticles (MnFe_2_O_4_) as MRI CAs

Finally, although not being the main subject of this review, the use of Mn(II) ferrite nanoparticles (MnFe_2_O_4_) as MRI-based multimodal imaging and theranostic agents is briefly discussed using a representative example. MnFe_2_O_4_ NPs have mixed spinel structures, where the Mn^2+^ and Fe^3+^ cations occupy A (tetrahedral) and B (octahedral) sites formed by the face-centered arrangement of oxygen anions [Mn^2+^_1−i_ Fe^3+^_i_]_A_[Mn^2+^_i_ Fe^3+^_2−i_ ]_B_O_4_, where i is the inversion parameter. In normal spinels (i = 0), all of the Mn^2+^ are in tetrahedral sites and in inverse spinels (i = 1) they occupy the octahedral sites. The magnetization of these NPs increases for low degree of inversion, depending on the synthetic methods. As the normal spinel is the thermodynamically most stable form of MnFe_2_O_4_ NPs, they are superparamagnetic with a very high saturation magnetization (M_S_), making them useful as MRI CAs, particularly as negative (T_2w_) agents. As these NPs are effective as heater sources under oscillating magnetic fields, they are also useful for magnetic hyperthermia (MHT)-mediated cancer therapy [75,76].

A recent example of such applications was recently reported by Shabalkin et al., who produced by hydrothermal synthesis core-shell ZnFe_2_O_4_@MnFe_2_O_4_ NPs with 5.5 nm core diameter and shell thicknesses of 0.5 (ZM0.5) and 1.7 nm (ZM3). Their water suspensions are stable upon citrate coating, and the ZM3 NPs showed values of r_1_ = 2.1 mM^−1^s^−1^ and r_2_ = 63.4 mM^−1^s^−1^ (r_2_/r_1_ = 30.2) at 1.5 T, allowing T_1_–T_2_ dual-mode contrast properties. The high M_S_ = 47.61 emu g^−1^ of ZM3 NPs led to a high specific absorption rate of 8 W g^−1^ in the presence of a 100 Oe alternating magnetic field with a frequency of 75 kHz, resulting in heating up to 42 °C within a few seconds. A γ ray radiotherapy (RT) study with HCT116 cells after uptake of the NPs demonstrated their radiosensitizer properties, with a significant dose factor enhancement (about 40%) for both types of NP. Thus, these multifunctional theranostic NPs showed excellent in vitro cell properties of dual-mode T_1_–T_2_ MRI contrast ability for detailed diagnosis and MHT/RT potential for complex synergistic treatment [77].

### 3.2. Mn(III) Systems

Mn(III) oxide nanosystems can be classified into Mn(III) oxo clusters and Mn_3_O_4_-based NPs.

#### 3.2.1. Mn(III)-Based Oxo Clusters and Derivatives

The properties of Mn(III)-based oxo clusters as MRI CAs, such as the single molecule magnet Mn-12 ([Mn_12_O_12_(O_2_CCH_3_)_16_(H_2_O)_4_]), have been described in detail [78,79,80]. Mn-12 undergoes ligand exchange easily, which only modestly affects the properties of the core: This property provides a synthetic tool to modify or graft the intact cluster to nanostructured materials [81]. As an example, Mn-_12_ was coordinated to a polymer bead surface, or encapsulated by an assembly to be used as MRI CAs [78,82]. While the cluster precipitates in water, it gains stability by surface attachment e.g., of acetate, which affects the r_1_ value of Mn-12.

Incorporation of Fe(III) into Mn-12 to produce Mn_8_Fe_4_O_12_ (O_2_CCH_3_)_16_(H_2_O)_4_ (or Mn_8_Fe_4_) increases the spin state (8.58 μ_eff_/molecule) due to the substitution of Mn(III) (S = 2) by Fe(III) (S = 5/2). The iron substitution increases the cluster stability and solubility in water [83]. Like Mn-12, these clusters undergo ligand exchange reactions, so copolymers of Mn_8_Fe_4_O_12_(VBA)_16_ (VBA = vinyl benzoic acid) with styrene were used to prepare magnetic nanobeads [84]. The mixed-metal-oxo clusters, Mn_8_Fe_4_O_12_ (L)_16_(H_2_O)_4_ (L = O_2_CCH_3_ or VBA) were studied as potential MRI CAs. The clusters alone, with r_1_ = 2.38 mM^−1^ s^−1^ and r_2_ = 29.5 mM^−1^ s^−1^ (7 T), showed promise as T_2_ CAs in phantom T_1w_ and T_2w_ images, with low cytotoxicity towards two human prostate cancer cell lines, DU-145 and LNCap. The Mn_8_Fe_4_O_12_(VBA)_16_ cluster was copolymerized with styrene and the resulting Mn_8_Fe_4_O_12_-co-polystyrene copolymer (Mn_8_Fe_4_-co-PS) nanobeads (70.0 nm size, TEM) formed water stable dispersions (84 nm hydrodynamic size, DLS), with r_1_ = 3.37 mM^−1^ s^−1^ and r_2_ = 26.65 mM^−1^ s^−1^. Biocompatible dextran-coated Mn_8_Fe_4_O_12_-co-styrene copolymer nanobeads have a small increase in r_1_ from 2.38 for the cluster to 3.37 mM^−1^ s^−1^ for the nanobeads, despite the increased diameter, and an increased r_1_ from 3.37 for uncoated to 6.72 mM^−1^ s^−1^ for the dextran-coated nanobeads, possibly due to the hydrodynamic size increase to 165 nm or increased coat hydrophilicity. The r_2_ values also increased upon dextran coating from 11.07 for uncoated to 26.00 mM^−1^ s^−1^ for coated nanobeads. The r_2_/r_1_ = 3.87 value for dextran-coated nanobeads showed that both types of nanobeads can be used as T_1_ CAs in the phantom MRI images [85].

In a continuation of the previous studies, nonporous Mn_8_Fe_4_-co-PS nanobeads were compared with Mn_8_Fe_4_-co-PS-IO (IO = iso-octane, 1.65% by weight) nanobeads. The presence of the IO branched hydrophobic moiety led to the appearance of voids in pure polystyrene nanobeads, which enhanced the porosity and the water flux through the carrier. These porous nanobeads were homogenous in size, with an average diameter of 72.7 nm (TEM) and average hydrodynamic diameter of 86.0 nm (DLS). The porous nature of the nanobeads was proven by nitrogen gas adsorption isotherms, showing that the beads had an increased nitrogen uptake relative to the nonporous beads. The porous beads had a higher relaxivity (r_1_ = 3.8 mM^−1^ s^−1^) than the nonporous beads (r_1_ = 5.2 mM^−1^ s^−1^) at 9.4 T, due to their higher water permeability and faster water exchange. The r_2_ of 11.9 mM^−1^ s^−1^ in Mn_8_Fe_4_-co-PS beads increased to r_2_ = 50.1 mM^−1^ s^−1^ for the porous Mn_8_Fe_4_-co-PS-IO beads, due to greater water accessibility in the second. Despite the high r_2_/r_1_ ratio (∼10), the positive contrast enhancing properties of these beads were measured in T_1w_ phantom MR images, also with a strong T_2_ effect resulting from the high effective magnetic moment of the clusters. The porous beads are stable in 10% human serum, showed no metal leaching and low cytotoxicity towards PDAC cells. Dye-tagged porous nanobeads internalized into PC3 cells by nonspecific endocytosis, localizing predominantly in the perinuclear region, including the Golgi apparatus and the endoplasmic reticulum, as shown by confocal laser scanning microscopy. Upon i.v. injection of the beads in healthy FVB mice, they were predominantly taken up by mononuclear phagocyte system (MPS)-rich organs, such as the liver and spleen. T_1w_ and T_2w_ MRI images of a BALB/c mouse following i.v. tail injection of the porous nanobeads highlighted the highest contrast in the spleen, liver, and kidney, supporting a mixed renal and hepatic clearance. The nanobeads remained intact in vivo, as demonstrated by synchrotron X-ray fluorescence microscopy. The experimental data illustrate the potential of the porous Mn_8_Fe_4_-co-PS-IO nanobeads as DMCAs [86].

#### 3.2.2. Mn_3_O_4_ Nanoparticles

The synthesis, characterization, chemical properties and performance of Mn_3_O_4_-based NPs as targeted, responsive, uni- and multimodal MRI CAs, as well as their theranostic applications, have been described in several recent reviews [9,44,45,87,88]. In this publication, some recent representative examples of each type of application will be presented, and the properties of some of them are summarized in Table 3.

1. Mn_3_O_4_-Based NPs as MRI CAs

Like MnO NPs, Mn_3_O_4_ NPs have also been extensively developed as MRI CAs. Hyeon et al. first reported an easy, low-temperature synthesis of Mn_3_O_4_ nanocrystals of various shapes and sizes in nonpolar solvents in the presence of surfactants. The nanocrystals were characterized using TEM and powder XRD. Different experimental conditions, such as precursors, surfactants, and water injection temperature, were able to control the size and shape of the nanocrystals. Their observed shapes included spherical (sizes from 5.5 to 15 nm), nanoplates (5 nm thickness and side dimensions of 9, 15 and 22 nm), and asymmetric nanowires and nanokytes. Water-dispersible 9 nm sized Mn_3_O_4_ nanoplates showed relaxivities r_1_ = 0.13 mM^−1^ s^−1^ and r_2_ = 0.55 mM^−1^ s^−1^ at 1.5 T (r_2_/r_1_ = 4.2), that, despite being small and with a high r_2_/r_1_ ratio, demonstrated their potential application as T_1_ MRI CAs [89].

Contrary to MnO NPs, with only Mn^2+^ ions, Mn_3_O_4_ (Mn_2_O_3_.MnO) NPs contain Mn^2+^ and Mn^3+^ ions. Mn_3_O_4_ has a spinel structure with O atoms closely packed with Mn^2+^ in the tetrahedral sites and Mn^3+^ in the octahedral sites (Mn^2+^[Mn^3+^]_2_O_4_^2−^). Compared with Mn^2+^ ions, Mn^3+^ tends to induce lower T_1_ relaxation due to fewer unpaired electrons (and thus smaller effective magnetic moment) and a shorter electron spin relaxation time than Mn^2+^ (Table 1). However, Mn^3+^ ions can be reduced to Mn^2+^ in an intracellular reducing environment by GSH, increasing the r_1_ relaxivity and producing redox-activated T_1_ MRI CAs.

As in the case of MnO NPs, described in Section 3.1, only the surface ions of Mn_3_O_4_ NPs in contact with bulk water molecules are responsible for T_1_ contrast enhancement, and their number is highly related to the surface-to-volume ratio (S/V) of the NPs, which depends on their shape. Yeh et al. reported a study on sub-10-nm nanospheres, nanoplates, and nanocubes of MnO NPs passivated by a layer of Mn_3_O_4_. The reason for this passivation is the decrease of their r_1_ and r_2_ values by pH-dependent Mn^2+^→ Mn^3+^(Mn^4+^) oxidation. The r_1_ for nanoplates (2.06 mM^−1^ s^−1^) was higher than for nanospheres (1.31 mM^−1^ s^−1^) (both at 3 T) of Mn_3_O_4_@PSS dispersions (PSS = polystyrene sulfonate), demonstrating a direct correlation between r_1_ and the S/V ratios of the nanostructured morphologies (0.73 vs. 0.61, respectively). However, their r_2_/r_1_ values of 4.9 were still too high. A 139% signal increase was found in T_1w_ MRI images of Mn_3_O_4_ nanoplate-treated A549 lung cancer cells compared with untreated cells [90].

The synthesis of uniform, thermodynamically controlled cubic, octahedral, and spherical Mn_3_O_4_ NPs by thermal decomposition of a Mn^2+^-oleate complex was reported. The smallest 3 nm-size nanospheres showed the highest r_1_ = 2.38 mm^−1^ s^−1^, due to their highest S/V ratio. Synthesized MnO nanocrystals transformed to either Mn_3_O_4_ or MnO@Mn_3_O_4_ core-shell NPs upon air oxidation, depending on their size and shape, which changed their magnetic behavior, forming an antiferromagnetic core and a ferrimagnetic shell [91].

The synthetic conditions for formation of MnO, Mn_3_O_4_ or both simultaneously by pyrolysis of Mn(II) acetate in 1-octadecene in the presence of oleylamine and oleic acid have been studied on the basis of the kinetics of coordination between Mn^2+^ and oleic acid/oleylamine. It was concluded that Mn^2+^-oleylamine coordination favors the formation of Mn_3_O_4_ nanocrystals due to the low electronegativity of the oleylamine nitrogen atom. Conversely, Mn^2+^ oleate coordination can inhibit the oxidation of Mn^2+^ due to the high electronegativity of the oleate oxygen. Thus, the selective synthesis of MnO and Mn_3_O_4_ nanocrystals with uniform shapes was allowed by balancing the Mn^2+^ coordination by oleic acid and oleylamine [92].

The oxidation of MnO NPs (MONs) into hollow Mn_3_O_4_ (HMONs), which were used as T_1_ and T_2_ MRI CAs and vehicles for drug (DOX) cell labeling, has been reported. In vivo T_1w_ and T_2w_ MRI of a mouse brain upon local injection of HMONs showed a positive and negative contrast on the injection site, showing that these NPs have potential as dual contrast agents for both T_1w_ and T_2w_ MRI [93].

Poon et al. studied the effect of different synthetic procedures and experimental conditions on the size distribution, chemical composition, degradation and corresponding magnetic and r_1_ and r_2_ NMR relaxivities of manganese oxide NPs (MONs). The MONs were synthesized in anoxic (N_2_) and normoxic (air) environments, using the metallic precursors Mn(II)-oleate and Mn(II) acetylacetonate. The four types of NPs obtained were labeled MONP-N_2_, MONP-air, MONP-8 (N_2_) and MONP-6 (air). Their sizes, structures and chemical compositions were studied by TEM, HR-TEM, XRD and X-ray photoelectron spectroscopy (XPS). The sizes of MONP-N2 (10 ± 2 nm), MONP-air (9 ± 2 nm), MONP-8 (N_2_) (8 ± 1 nm) and MONP-6 (air) (6 ± 1 nm) decreased steadily along the series. Their chemical compositions were found to be Mn_3_O_4_, except for MONP-N_2_, with a core–shell Mn_3_O_4_–MnO structure. The magnetic properties of different MONPs were studied by magnetometry. Bulk Mn_3_O_4_ was ferrimagnetic with a Curie temperature T_C_ = 42 K, and MnO was antiferromagnetic with a bulk Néel temperature T_N_ = 118 K. MONP-N_2_, MONP-air and MONP-8 (N_2_) were superparamagnetic at room temperature and ferromagnetic below 40 K. To study the potential of these MONPs as MRI CAs and drug delivery systems, the MONs were functionalized with the drug L-3,4-dihydroxyphenylalanine (L-DOPA), which stabilized the NPs in water. Their r_1_ and r_2_ values (0.5 T) in aqueous solutions were measured in water immediately after dispersion and after 24 h aging. The values changed with time: MONP-N_2_ (r_1_ = 1.8 →1.2, r_2_ = 4.0 → 2.4), MONP-air (r_1_ = 0.2 → 1.2, r_2_ = 1.1 → 3.3), MONP-8 (N_2_) (r_1_ = 6.5 → 12.2, r_2_ = 17.4 → 24.1) and MONP-6 (air) (r_1_ = 1.9 → 3.1, r_2_ = 2.9 → 6.1), with r_2_/r_1_ values in the 1.5–2.7 range, except for the initial value of 5.5 for MONP-air. These values were interpreted in terms of differences in S/V ratios, water exposure of the ions, composition (Mn(II) %), and degradation of the NPs. A preliminary cell viability study with human fibroblasts showed no cytotoxicity of the MONs-L-DOPA conjugates, which have potential as T_1_ MRI CAs and for L-DOPA drug delivery in the treatment of Parkinson’s disease [94].

Other researchers have investigated Mn_3_O_4_ nanocubes with high S/V ratios, nanosheets, and several different non-spherical morphologies with high relaxivities, providing better knowledge of non-spherical Mn(III)-based MRI CAs.

Many hybrid Mn_3_O_4_-based NPs have been studied as potential T_1_ CAs with their surfaces coated or modified using organic and inorganic molecules. The surface coating avoids colloidal instability in water, improves their biocompatibility, and also may improve their T_1_ relaxivity, making them usable as in vivo MRI CAs. The most commonly employed modification approach involves the encapsulation of hydrophobic NPs by polymers, such as PEG, PEI, PSS, PAA and polydopamine (PDA), but silica inorganic coatings are also common.

An example of PEG-modified systems is the design by Deka et al. of Mn_3_O_4_ NPs encapsulated in a mesoporous 3D carbon framework (CF) functionalized by PEG as a T_1_ MRI CA. The Mn_3_O_4_@CF NPs had an average size of ∼50 nm, as measured by TEM. Magnetization curves, measured in the temperature range of 5–350 K at an applied field H = 100 Oe, showed a phase transition from paramagnetic to ferromagnetic at 47 K, corresponding to the Curie temperature (T_C_) of Mn_3_O_4_. PEG functionalization was responsible for the water solubility and stability of the NPs, while the CF avoided metal ion exposure and thus provided excellent biocompatibility. Their in vitro cytotoxicity was negligible up to a 0.5 mM concentration, as obtained by an MTT viability assay of the RAW 264.7 cell line. The r_1_ (3.5 mM^−1^ s^−1^ at 1.41 T) of the NPs was rationalized by the access of the Mn_3_O_4_ core to bulk water via exchange through the CF pores. Thus, this system has good potential as an in vivo T_1_ MRI CA [95].

**Figure 2:**
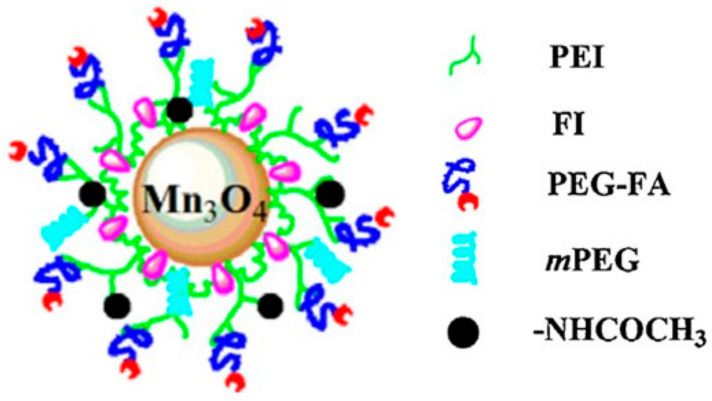
Structure of the Mn_3_O_4_-PEI-Ac-FI-mPEG-(PEG-FA) NPs. Adapted from Ref. [96].

Another commonly used type of polymer is PEI. For example, Luo et al. reported monodispersed acylated PEI-coated Mn_3_O_4_ NPs sequentially conjugated with fluorescein isothiocyanate (FI), folic acid (FA)-linked PEG and PEG monomethyl ether (Mn_3_O_4_-PEI-Ac-FI-mPEG-PEG-FA NPs), which were tested as tumor-targeted CAs for in vivo MRI. The NPs were characterized by TEM (8.1 nm) and XRD. They were water dispersible and colloidally stable due to the PEG coating, with a hydrodynamic size of 571.0 nm and a zeta potential of +18.8 mV. Their r_1_ of 0.57 mM^−1^ s^−1^ indicated that they could be used as a positive CA in T_1w_ MRI applications. The NPs showed no cytotoxicity (MTT assay) and were able to target FA receptors overexpressed by KB cancer cells in vitro, as verified by flow cytometry and confocal microscopy. The effective use of the NPs as FA-targeted positive CAs in T_1w_ MRI applications was verified in vitro with KB cancer cells and in vivo using a xenografted KB tumor mouse model (Figure 2) [96].

Although both PEG and PEI modification strategies are common, they create a thick coating shell of hydrophobic hydrocarbon that usually hinders the access of water to the Mn_3_O_4_ core, leading to relatively low r_1_ values. A different approach is based on the use of hydrophilic coatings, such as alginate (AG) nanogels. Sun et al. reported Mn_3_O_4_-PEI NPs loaded with hybrid alginate (AG) (AG/PEI-Mn_3_O_4_), with a 141.6 nm size (TEM) and a hydrodynamic size of 219.1 nm (DLS), which had an r_1_ of 26.12 mM^−1^·s^−1^ at 0.5 T, nearly 20 times greater than that of the PEI-Mn_3_O_4_ NPs, due to their much-improved access to bulk water. They also showed a prolonged blood circulation time and improved tumor in vivo MRI detection compared to the PEI-Mn_3_O_4_ NPs [97].

Another alternative is the use of small molecules such as sodium citrate (SC) on hydrophobic NP surfaces. However, synthesis experimental conditions may result in the total oxidation of Mn^2+^ to Mn^3+^ ions (Mn_2_O_3_), which strongly reduces the obtainable r_1_. Therefore, an optimized procedure for surface modification was needed to increase the r_1_ value of MONs.

Lee et al. investigated the impact of various end-capping ligands—such as carboxylate, alcohol, mercaptan, and amine, each possessing different anchoring groups—to optimize the surface functionalization of HMONs and enhance their r_1_ values. HMONs were functionalized with dopamine-mPEG (CC-PEG), octadecyl-phosphonic acid (OP), bisphosphonate-PEG (BP-PEG), oleic acid (OA), oleylamine (OAm), oleyl alcohol (OH) and 1-hexadecanethiol (SH) ligands, resulting in the water-dispersible CC-PEG-HMON, OP-PL-HMON, BP-PEG-HMON, OA-PL-HMON, OAm-PL-HMON, OH-PL-HMON, and SH-PL-HMON. The capped HMONs were characterized in the solid state by TEM, XRD, FT-IR and EDS. The DLS-based hydrodynamic sizes of aqueous suspensions of those derivatives were found in the 270−310 nm range. The r_1_ and r_2_ values (at 3 T) of water suspensions of the HMONs revealed that OA-PL-HMON had the highest relaxivities (r_1_ = 1.10 mM^−1^ s^−1^, r_2_ = 9.2 mM^−1^ s^−1^). At the same time, among all the systems studied, the HMONs surface-capped with the carboxylate-anchored ligand OA had a significantly increased magnetization. This increase was used to explain the enhanced r_1_ value, unlike previous explanations based on the water accessibility of surface Mn^2+^ ions. The HMONs showed no significant cytotoxicity to primary neuron cells of isolated mouse embryos. Finally, in vivo MRI imaging of BALB/c nude mice (7 T) upon brain injection of HMON, OA-PL-HMON and OAm-PL-HMON was performed. T_1w_ images showed positive contrast at the injection site in all cases, which was brightest with OA-PL-HMON and remained after 8 h, validating its highest r_1_ value. T_2w_ MRI showed the expected negative contrast in all cases but persisted for shorter times than the positive contrast [98].

Wang et al. developed multifunctional antifouling zwitterion-functionalized Mn_3_O_4_ NPs modified with folic acid (FA) as a targeting agent for tumor T_1w_ MRI, which was coated with polydopamine (PDA), labeled with fluorescent rhodamine B, and finally covered with zwitterions of L-lysine (Lys). These Mn_3_O_4_−PDA−RB−FA−Lys NPs were characterized using FT-IR and UV−vis spectroscopies, TGA and TEM (average Mn_3_O_4_ core average size of 3.89 nm). In aqueous suspensions, the NPs had a hydrodynamic diameter of 198.7 nm (DLS) and a negative ζ-potential of −24.90 mV, which caused them to be highly dispersible and colloidally stable in water, PBS and cell culture medium. They showed a very high r_1_ of 89.30 mM^−1^ s^−1^ (0.5 T) due to the PDA surface coating and modification of zwitterionic Lys, which increase the NPs volume (longer τ_R_) without affecting their water accessibility. The NPs showed good cytocompatibility to CCK-8 cells and enabled targeted tumor visualization in T_1w_ MRI (3 T) of a xenografted male BALB/c nude mouse model subcutaneously injected with KB cells, owing to the linked FA ligands [99].

Finally, Guo et.al. reported a liver T_1w_ MRI CA consisting of ultrathin M Mn_3_O_4_ nanosheets supported on caramelized carbonaceous nanospheres (Mn_3_O_4_-CNPs) with good biocompatibility and a high in vitro relaxivity (r_1_ = 11.6 mM^−1^ s^−1^ at 3 T). Their in vivo MRI assessment was performed in normal, healthy Balb/c mice before and at 15 min, 30 min, 1 h, 2 h, 4 h,12 h, and 24 h after administration of the nanospheres. The liver signal intensity (SI) of mice increased by 50.1% 4 h after injection of the CA in T_1w_ MRI images (3 T) as a result of being taken up by Kupffer cells. Therefore, the Mn_3_O_4_-CNPs are potential biocompatible liver MRI CAs [100].

2. Mn_3_O_4_-based NPs as Multimodal MRI CAs

**Figure 3:**
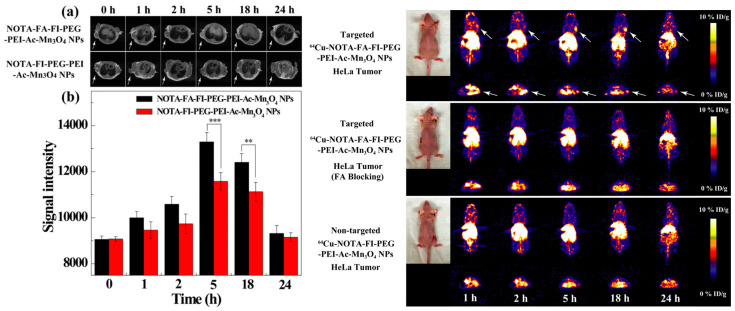
Left: In vivo T_1w_ MR images (**a**) and signal intensity (**b**) of HeLa tumors post i.v. administration of the NOTA−FA−FI−PEG−PEI−Ac−Mn_3_O_4_ NPs or the NOTA−FI−PEG−PEI−Ac−Mn_3_O_4_ NPs (500 mg Mn, in 0.2 mL saline) at different times. Tumors are indicated by white arrows. Mean values were compared via one-way analysis of variance and Student’s *t*-test and data were marked with (**) for *p* < 0.01, and (***) for *p* < 0.001, respectively.; right: micro-PET images of nude mice bearing HeLa xenografted tumors at different times post i.v. injection of the ^64^Cu−NOTA−FA−FI−PEG−PEI−Ac−Mn_3_O_4_ NPs (targeted NPs), the ^64^Cu−NOTA−FA−FI−PEG−PEI−Ac−Mn_3_O_4_ NPs with FA blocking, and the ^64^Cu−NOTA−FI−PEG−PEI−Ac−Mn_3_O_4_ NPs (non-targeted NPs). The whole-body coronal (top) and transverse (bottom) micro-PET images of nude mice bearing HeLa xenografted tumors are presented. Tumors are indicated by arrows. Reproduced with permission from Ref. [101], Copyright (2018) by the American Chemical Society.

Zhu et al. reported a nanoplatform consisting of PEG-FA-FI (fluorescein isothiocyanate)-PEI-coated Mn_3_O_4_ NPs (Figure 2), surface-labeled with ^64^Cu(NOTA) chelates, as a targeted PET/T_1_-MRI bimodal imaging probe (^64^Cu(NOTA)/PEG−FA-FI-PEI− Mn_3_O_4_). The combined high sensitivity of PET and the superb spatial resolution of MRI are complementary for dual-mode clinical imaging of cancer. The obtained nanoprobes were fully characterized by TEM (average diameter 7.29 nm), XRD and FTIR. In aqueous solutions, their hydrodynamic size was much larger (476.5 nm), as obtained by DLS, indicating NP clustering in solution due to near-zero ζ potential. The NPs were stable in water solution, with r_1_ = 0.996 mM^−1^ s^−1^ at 0.5 T. They had no in vitro cytotoxicity (MTT test) and showed target specificity to folate receptors (FR) overexpressed in HeLa tumor cells, as observed by confocal microscopy and flow cytometry. In vivo microPET images of nude mice bearing HeLa xenografted tumors showed good tumor tracer uptake, with a tumor-to-muscle (T/M) ratio of 5.35 at 18 h post-injection (p.i.) of the NPs, which decreased to 2.78 at 18 h p.i. in the FR-blocked tumors. They also caused positive tumor contrast in T_1w_ MRI images (1.5 T) when applied in PET/MRI of small animals. When compared with HeLa tumors blocked by folate receptors (FR), ^64^Cu-labeled Mn_3_O_4_ NPs reported an improved uptake of the tracer in HeLa tumors expressing FR 18 h post-injection. Thus, the nanoplatform proved to be an effective tumor-targeted bimodal PET/MRI probe for animal studies (Figure 3) [101].

T_1_/T_2_ MRI DMCAs have the advantage of providing complementary information, which improves the accuracy of the images, but most of them have the disadvantage of being always “ON”, providing MRI contrast that is independent of their interaction with target cells or biomarkers, resulting in low target-to-background ratios. Kim et al. designed a redox-responsive activatable nanostar shell (RANS) operating as a magnetic relaxation switch (MGRS) that activates a T_1_/T_2_ DMCA. It consists of NPs with a superparamagnetic Fe_3_O_4_ core and a paramagnetic Mn_3_O_4_ shell (Fe_3_O_4_@Mn_3_O_4_). The Mn_3_O_4_ shell causes weak T_1_ relaxation and attenuates the strong T_2_ relaxation effect of the Fe_3_O_4_ core. It also acts as a redox switch that can be activated by GSH in intracellular reducing environments. By this process, high concentrations of free Mn^2+^ ions are generated, and Fe_3_O_4_ becomes free to interact with the water protons. Thus, the system switches from lower r_1_ (2.4 mM^−1^.s^−1^) and r_2_ (92.2 mM^−1^.s^−1^) to much higher r_1_ (16.1 mM^−1^.s^−1^) and r_2_ (258.6 mM^−1^.s^−1^) values, measured at 1.5 T, upon reaction with GSH in vitro (on/ON redox switch). The NPs have a 13.3 nm size (obtained by TEM) and show no cytotoxic effects (MTT test) and fast uptake in MKN-45 cells. The NPs worked as T_1_/T_2_ MRI DMCA both in vitro (1.5 T) by adding the NPs to MKN-45 cells and in vivo using MRI (3.0 T) of BALB/c-nude mice injected in the proximal thigh with the NPs, observing in both cases an efficient transition of OFF/ON dual contrast effect. Thus, these NPs are able to act as DMCAs, illustrated by effective passive tumor targeting for T_1w_ and T_2w_ MRI of tumor-bearing mice [102].

Yang et al. developed monodisperse core-shell silica-coated Mn_3_O_4_ NPs, whose silica shell was aminated through salinization with APS and covalently conjugated to a fluorescent dye, rhodamine B isothiocyanate (RBITC), and folate (FA). The resulting Mn_3_O_4_ @SiO_2_(RBITC)–FA core–shell nanocomposites were characterized by XRD, FT-IR, TEM (mean diameter of ~35 nm), and UV-vis and fluorescence spectroscopy. The NPs were water-stable, with an r_1_ of 0.50 mM^−1^.s^−1^ (0.5 T) and 0.47 mM mM^−1^.s^−1^ (3.0 T), and biocompatible (for Mn concentrations below 50 mg mL^−1^), as shown by an MTT cytotoxicity assay on HeLa cells. A combination of confocal microscopy, flow cytometry and in vitro MRI proved that the NPs could target cancer cells overexpressing FA receptors (FARs) specifically, suggesting that these NPs were useful in bimodal T_1w_ MRI and fluorescence imaging [103]. Ji et al. successfully obtained nanocomposites (NCs) composed of porous Mn_3_O_4_ nanostructures decorated with very small PtO_x_ NPs (Mn_3_O_4_/PtO_x_) with a high r_1_ (20.48 mM^−1^.s^−1^) and a computed tomography attenuation value much better than the commercial product iopromide. These led to outstanding T_1w_ MRI and CT imaging performances of these NCs upon i.v. injection in tumor-bearing mice [104]. Finally, Li and colleagues reported a tumor-targeted nanoprobe caging relaxivity-silent Mn_3_O_4_ nanocrystals (12.2 nm) within transferrin nanocages (Mn_3_O_4_-Tf) targeted to TfR1 receptor overexpressed in HT-19 colon tumor cells. This probe rapidly decomposed in the tumor cells in the presence of lysosomal acid pH and GSH, producing high r1 Mn^2+^ ions, which were sequentially captured/retained and re-amplified by extravascular albumin in the tumor in vivo, as detected by T_1w_ MRI of HT-29 tumor Balb/c mice, as well as SPECT-CT using ^99m^Tc-labeled probes and OI and PAI of CyT5-labeled probes [105].

3. Mn_3_O_4_-Based as MRI-Guided Theranostic Agents

The development of theranostic methods, combining diagnostic and therapeutic tools ideally in a single nanoplatform, is of great importance for the simultaneous diagnostics and specific therapy of cancer. A variety of nanotechnology approaches have been used by many research teams to contribute to the development of Mn_3_O_4_-based theranostic agents. Here, some representative examples are described.

Wang and colleagues developed hydrophilic amine-terminated Mn_3_O_4_ nanolids as a redox-mediated multifunctional mesoporous silica-based nanotheranostic system. Their assembly used functionalization of the mesoporous silica nanoparticle surface with carboxylate groups (MSN-COOH), which bound camptothecin (CPT) at its nanochannels. After this, Mn_3_O_4_-NH_2_ nanolids were obtained by treating hydrothermally synthesized Mn_3_O_4_ NPs with 3-aminopropyltriethoxysilane (APTES), which were then capped to the CPT-loaded MSN-COOH structure. The water-soluble Mn_3_O_4_@MSN@CPT nanolids had a relaxivity r_1_ = 13.39 mM^−1^ s^−1^ at 3 T and were non-toxic to pancreatic cancer cells (BxPC-3) loaded with the nanolids. Exposure of the lids to the intracellular reducing environment resulted in their dissolution by reaction with GSH, causing the intelligent release of the CPT drug. Upon the redox reaction with GSH, the Mn_3_O_4_ NPs dissociated and released free Mn^2+^, which doubled the r1 relaxivity (r_1_ = 25.17 mM^−1^ s^−1^) (on/ON redox switch). In vivo evaluation of the system by MRI (3 T) showed signal enhancement in both the kidney and liver in T_1w_ images upon tail vein injection of mice with the nanolids, also allowing MRI tracking of the therapy feedback [106].

Nafiujjaman et al. developed ternary hybrid nanoprobes consisting of Mn_3_O_4_ NPs conjugated to graphene quantum dots (GQDs) through polydopamine (PDA) and thiol-amine, GQD-PDA-Mn_3_O_4_ NPs, as dual (MRI/fluorescence) imaging-guided photodynamic therapy (PDT) agents. The NP morphology was characterized by FT-IR, TEM (mean diameter around 100 nm), and UV-vis and fluorescence spectroscopy. This hybrid system was stable in solution due to the stability of the linker, which reduced the quenching of the GQD red fluorescence by Mn_3_O_4_ by keeping them far apart. While these NPs were non-cytotoxic upon uptake by A549 and MDCK cells in dark conditions, 670 nm laser irradiation triggered GQDs to generate effective fluorescent emission and ROS, killing the cancer cells through a phototherapeutic effect. The relaxivity of the NPs in vitro (r_1_ = 3.5 mM^−1^ s^−1^ at 3 T) indicates their potential as positive MRI CAs, which was confirmed by positive contrast in the tumor region of in vivo T_1w_ MRI (4.5 T) images of A549 tumor-bearing mice i.v. injected with the NPs. The tumor accumulation of the NPs also led to PDT-induced tumor regression upon exposure to the 670 nm laser, as shown by in vivo NIR fluorescent and MRI images. Thus, the hybrid NPs exhibited excellent theranostic OI/MRI/PDT capability [107].

**Figure 4:**
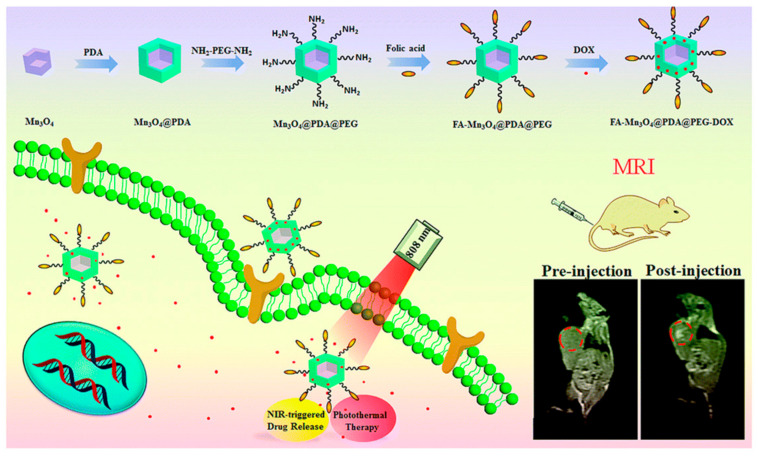
Scheme illustrating the core/shell nanotheranostic Mn_3_O_4_@PDA NP design and synthesis for MRI guided synergetic chemo-/photothermal therapy. Reproduced from Ref. [108].

Another nanotheranostic system was designed by Ding and colleagues for MRI-guided combinatorial cancer chemo-/photothermal therapy (PTT). It consisted of Mn_3_O_4_ NPs with the surface covered with PDA, which was conjugated to drug (DOX)-loaded PEG-FA. The (FA(DOX)-Mn_3_O_4_@PDA@PEG) nanoplatform was characterized by TEM, XRD, FTIR, XPS and HAADF-STEM. The NPs showed excellent colloidal stability in water and strong NIR absorption due to the oxidation and self-polymerization of dopamine to PDA. After exposure to an 808 nm NIR laser, it displayed effective photothermal heating of the solution, adequate for PTT. It could also efficiently load the chemotherapeutic DOX drug, which was taken up by MCF-7 cells, as shown by confocal microscopy, and showed no cytotoxicity. Therefore, the PDA can provide biocompatibility to the NPs and could act as an agent for PTT photothermal conversion and an anti-cancer drug carrier. The NPs, with a high r_1_ = 14.47 mM^−1^ s^−1^, were an excellent MRI CA in vitro. These properties were confirmed in vivo. T_1w_ MRI (3 T) images of tumor-bearing Balb/c mice showed brightening of the whole tumor area 36 h after i.v. injection of the NPs, demonstrating accumulation of a large amount of the NPs in the tumor through the active targeting capacity of the FA vector as well as the enhanced permeability and retention (EPR) effect occurring in the tumor vessels. The combined chemo-/PTT therapeutic effects of the NPs were verified by the effective in vivo tumor inhibition in H22 tumor-bearing mice under 808 nm NIR irradiation, which triggered the DOX drug release. Thus, the system was quite effective for cancer treatment based on MRI-guided synergetic chemo-/PTT (Figure 4) [108].

Liu et al. developed a new inorganic nanosystem for T_1_/T_2_ MRI-guided PTT. In order to avoid nanotoxicity, the authors designed EDTA- and BSA-capped Mn_3_O_4_ (Mn_3_O_4_-BSA-EDTA) NPs, which underwent controlled degradation by ascorbic acid. The NPs were characterized by TEM (50 nm size), XRD, ICP-MS and NIR-UV-vis spectroscopy and were well dispersed and stable in water, with a hydrodynamic size of 50 nm (DLS), and showed strong NIR absorption, responsible for a strong photothermal effect under 785 nm laser irradiation. The relaxivities of the NPs, r_1_ = 8.75 mM^−1^ s^−1^ and r2 = 40.09 mM^−1^ s^−1^ (0.5 T), were both high. Thus, the authors suggested that the NPs could act as T_1_/T_2_ dual-mode MRI CAs and also as PTT agents in vitro. The NPs were degraded by ascorbic acid to free ultra-small Mn_3_O_4_ NPs and free Mn^2+.^ The toxic Mn^2+^ could be captured by the BSA-EDTA coating of the NPs, avoiding the nanotoxicity of the inorganic NPs. In vivo confirmation of these properties included the observation of tumor bright signal enhancement and dark signal void in T_1w_ and T_2w_ MRI images of nude mice bearing HCT116 tumors. The photothermal effect was also verified in vivo through i.v. injection of the NPs into nude mice and recording of the temperature changes under a 785 nm laser by photothermal imaging, while the corresponding therapeutic effect was observed by the shrinking of the mice tumors. These experiments demonstrated that the NPs could be useful as T_1_/T_2_ MRI DMCAs for guided PTT in living systems [109].

Zhan et al. developed an optical/MRI dual-mode probe for in vivo bimodal imaging for guidance in sentinel lymph node (SLN) mapping, which could be used to estimate the metastatic stage of a tumor. It was based on PEG-coated Mn_3_O_4_ NPs conjugated to Cy7.5 dye (Mn_3_O_4_@PEG-Cy7.5 NPs). The NPs were characterized using several techniques, with a spherical shape of 8 nm size (TEM) and the Mn_3_O_4_ core having a single tetragonal crystalline phase (XRD). They had good colloidal stability in water, with a hydrodynamic diameter of 10 nm and a ζ potential of −10.3 mV, as obtained by DLS, as well as good biocompatibility. The fluorescence spectrum of the NPs, resulting from the Cy7.5 dye, proved the dye probe conjugation. The effectiveness of the NPs in aqueous solution as positive MRI and fluorescence imaging CAs was proven by an r_1_ = 0.53 mM^−1^ s^−1^ (7 T) and increased intensity in fluorescent images as the Mn and Cy7.5 concentrations were increased. The in vitro absence of toxicity and cell uptake of the NPs were verified in PC-3, A549, and HEPG2 cell models, using cell counting for the former and confocal microscopy and inductively coupled plasma-atomic emission spectrometry (ICP-AES) for the latter, showing their preferential localization in the nucleus cytoplasm periphery. These in vitro results were confirmed by in vivo fluorescence imaging (FI) and MRI (7 T). Upon i.v. injection of the NPs into healthy BALB/c mice, a fluorescence signal was detected in the liver, but subcutaneous injection led to visible labeling of the popliteal and sciatic lymph nodes at 0.5 h, 2 h and 12 h post-injection (p.i.) (n = 3). T_1w_ MRI images led to the same biodistribution of the NPs, through positive contrast in the same organs, which was confirmed by histological analysis of the organs. Thus, this work shows that the developed platform is valuable for dual imaging-based tumor metastasis diagnosis [110].

Arkaban et al. designed a nanocomposite for targeted imaging and therapeutics, consisting of AuNPs double-coated by MnCO_3_/Mn_3_O_4_ and PAA, as a bimodal X-ray CT and MRI contrast agent. Furthermore, by folic acid (FA) conjugation and DOX and fluorescent dye propidium iodide (PI) loading, it was also endowed with targeting, therapeutic and fluorescence properties (AuNPs@MnCO_3_/Mn_3_O_4_@PAA-FA (DOX and PI)). The system was characterized by TEM-EDXS, HAADF-STEM, EDXS-XRF and FT-IR. The loading with PI and the DOX anticancer drug inside the PAA-coated NPs, and the presence of FA at their surface, were monitored by UV/vis and fluorescence spectroscopy. The NPs had the following characteristics: (a) in vitro r_1_ of 12.62 and 1.32 mM^−1^ s^−1^ (1.5 T), at pHs of 5.5 and 7.4, showing potential as pH-sensitive MRI CAs in T_1W_ images; (b) the increased slopes of plots of Hounsfield unit (HU) vs. NP concentration, 23.22 and 32.63 HU L/g, obtained, respectively, for the PI-unloaded and loaded NPs, demonstrating the efficiency and the synergistic effect between the PI and AuNPs in the contrast enhancement of CT images in vitro; (c) targeting and intracellular delivery to fibroblast (L929) and breast cancer cells (4T1) shown by cytometry and fluorescence; (d) the enhanced in vivo anticancer effect of the DOX-loaded NPs vs. DOX, as shown by the growth inhibition of 4T1 breast tumors implanted in BALB/c mice; (e) the accumulation of the NPs in the tumor shown by biodistribution studies using elemental analysis of the organs by ICP-OES; and (f) the biocompatibility of the NPs based on blood biochemistry and histopathological data of mice injected with the NPs [111].

Finally, two quite recent examples are briefly described. Dar et al. used the triblock polymer Pluronic F-127 to enhance the biocompatibility of Au@Mn_3_O_4_ hybrid nanoflowers, which show great potential in T_1w_ MRI and PTT, as shown in vivo using 4T1 tumor Balb/c mice [112]. Foroushani et al. reported an integrated nanocomposite system comprising Mn_3_O_4_ NPs functionalized with PAA and the MOF ZIF-8, as a pH-sensitive drug delivery agent, and the anti-cancer drug methotrexate (MTX), operating as a tumor biomarker and a controlled drug delivery system with improved drug loading capacity (Mn_3_O_4_@PAA@ZIF-8/MTX), which was characterized in vitro using BT-474 and MCF-7 cancer cells [113].

It must be noted that among all the examples presented in this section based on Mn_3_O_4_ nanoparticles, some involve pH/redox responsiveness with release of toxic free Mn^2+^ ions as an approach to MRI signal enhancement [102,103,105,106,111], limiting their application to preclinical diagnosis.

**Table 3:** Overview of fundamental characteristics of some of the examples of Mn_2_O_3_-based NPs as MRI (and multimodal/theranostic) CAs covered in this section.

NPs Components	Imaging Modalities	Relaxivity (mM^−1^ s^−1^), 298 K, B_o_ (T)	Therapeutic Modalities	Cell/Animal Model	Reference
Mn_3_O_4_@CF	T_1_ MRI	r_1_ = 3.5 (1.41 T)	-	In vitro RAW 264.7 cells	[95]
Mn_3_O_4_-PEI-Ac-FI-mPEG-PEG-FA	T_1_ MRI	r_1_ = 0.57 (^a^)	-	In vitro KB cancer cells In vivo KB tumor mice	[96]
AG/PEI-Mn_3_O_4_	T_1_ MRI	r_1_ = 26.12 (0.5 T)	-	In vivo U87MG tumor mice	[97]
OA-PL-HMON	T_1_ MRI	r_1_ = 1.10; r_2_ = 9.2 (3.0 T)	-	In vivo BALB/c nude mice	[98]
Mn_3_O_4_-PDA-RB-FA-Lys	T_1_ MRI	r_1_ = 89.30 (0.5 T)	-	In vitro KB cells In vivo KB tumor Balb/c mice	[99]
Mn_3_O_4_-CNPs	T_1_ MRI	r_1_ = 11.6 (3.0 T)	-	In vivo BALB/c nude mice	[100]
^64^Cu(NOTA)/PEG−FA-FI-PEI−Mn_3_O_4_	T_1_ MRI/PET	r_1_ = 0.996 (0.5 T)	-	In vitro HeLa cells In vivo HeLa tumor mice	[101]
Fe_3_O_4_@Mn_3_O_4_	T_1_/T_2_ MRI	r_1_ = 2.4 →16.1 r_2_ = 92.2 →258.6 (1.5 T) redox responsive (GSH)	-	In vitro MKN-45 cells In vivo MKN-45 tumor Balb/c mice	[102]
Mn_3_O_4_@SiO_2_(RBITC)–FA	T_1_ MRI/OI	r_1_ = 0.50 mM (0.5 T) r_1_ = 0.47 (3.0 T)	-	In vitro HeLa cells	[103]
Mn_3_O_4_/PtO_x_	T_1_ MRI/CT	r_1_ = 20.48, r_2_/r_1_ = 1.46 (3.0 T)	-	In vivo tumor mice	[104]
Mn_3_O_4_-Tf (^99m^Tc/CyT.5)	T_1_ MRI/PAI/ SPECT-CT	r_1_ = 0.6 (pH 7.4) →3.5 (pH 5.0 (1.5 T) pH and redox (GSH) responsive	-	In vitro HT-29 colon tumor cells In vivo HT-29 tumor Balb/c mice	[105]
Mn_3_O_4_@MSN@CPT nanolids	T_1_ MRI/CT	r_1_ =13.39 → 25.17(3.0 T) redox responsive (GSH)	ChT(CPT)	In vitro BxPC-3 cells In vivo tumor mice	[106]
GQD-PDA-Mn_3_O_4_	T_1_ MRI/OI	r_1_ = 3.5 (3 T)	PDT	In vivo A549 tumor mice	[107]
FA(DOX)-Mn_3_O_4_@PDA@PEG	T_1_ MRI	r_1_ = 14.47 (3T)	ChT (DOX), PTT	In vitro MCF-7 cells In vivo MCF-7 and H22 tumor mice	[108]
Mn_3_O_4_-BSA-EDTA	T_1_/T_2_ MRI	r_1_ = 8.75; r_2_ = 40.09 (0.5 T)	PTT	In vivo HCT116 tumor mice	[109]
Mn_3_O_4_@PEG-Cy7.5	T_1_ MRI/OI	r_1_ = 0.53 (7T)	SDT	In vitro PC-3, A549, HEPG2 cells In vivo HEPG2 tumor Balb/c mice	[110]
AuNPs@MnCO_3_/Mn_3_O_4_@PAA-FA (DOX and PI)).	T_1_ MRI/CT	r_1_ = 12.62 (pH 5.5) r_1_ = 1.32 (pH = 7.0) pH responsive (1.5 T)	ChT (DOX)	In vitro 4T1 cells In vivo 4T1 tumor Balb/c mice	[111]
Au@ Mn_3_O_4_@F-127	T_1_ MRI	r_1_ = 5.74 (0.55 T)	PTT	In vivo 4T1 tumor Balb/c mice	[112]
Mn_3_O_4_@PAA@ZIF-8/MTX	T_1_ MRI	r_1_ = 5.74 (1.5 T)	ChT (MTX)	In vitro BT-474 and MCF-7 cells	[113]

^a^ Not indicated.

### 3.3. MnO_2_ Nanoparticles

Most MnO_2_-based NPs have been used for MRI-guided tumor theranostics, but some have been developed as MRI CAs for non-tumor cases. It should be emphasized that this type of NP often gives MRI signal enhancement upon release of Mn^2+^ ions that are known to be toxic; therefore, the authors discourage the use of MnO_2_-based NPs unless they are used for theranostic applications.

#### 3.3.1. MRI CAs of Non-Tumor Diseases

BSA–MnO_2_ nanoparticles (BM NPs) were developed for the permeability imaging of the blood–brain-barrier (BBB) and hemorrhage transformation (HT) prediction in acute ischemic stroke in vivo. The Mn(IV) valence state in the NPs was obtained by XPS spectrum, and their morphology (a monodisperse sphere-like geometry) and size (about 3 nm) were obtained by HRTEM. Their hydrodynamic size in aqueous suspensions (8.9 nm) and ζ potential (−20.0 mV) were responsible for high water solubility and long-term colloidal stability (at least 2 weeks). The presence of BSA in the NPs was shown by UV-Vis and FTIR. Along with these properties, their large r_1_ = 5.9 mM^−1^ s^−1^ (0.5 T), due to the presence of BSA, showed their potential as in vivo MRI CAs. Thus, middle cerebral artery occlusion (MCAO) rats with reperfusion after acute ischemic stroke were used to investigate the ability of the BM NPs for BBB permeability imaging. Since the NPs could not diffuse through the intact BBB, the increased intensity in T_1w_ images proved the permeability of the BBB to the NPs in the infarcted areas and allowed the stroke-related BBB damage to be visualized. A good prediction of the onset of HT in MCAO rats was shown by the peak intensity increase, the extended imaging duration, and the expanded imaging region indicated by the BM NPs in MRI. Thus, these NPs have high potential as MRI CAs for the noninvasive BBB permeability imaging in vivo, with impact on research of various neurological disorders and the treatment of stroke [114].

Wang et al. reported nanocomposites of albumin with pH-responsive MnO_2_ NPs (MnO_2_@BSA) for T_1w_ MRI of acute myocardial infarction (AMI) in rabbit models, using nanocomposites of albumin with non-pH-responsive Gd_2_O_3_ NPs (Gd_2_O_3_@BSA) as a positive control. Both nanocomposites had uniform spherical shapes with 20–30 nm diameter (TEM) and their aqueous suspensions had hydrodynamic diameters of 29 and 27 nm and ζ-potentials of −10.4 mV and −23.6 mV, respectively (DLS). MnO_2_@BSA released approximately 90% of Mn^2+^ at pH 5.0 within 72 h in a pH-triggered process under the acidic AMI microenvironment, while almost no Gd^3+^ was released from Gd_2_O_3_@BSA in the same conditions, indicating that these nanocomposites were stable in the AMI microenvironment. The MnO_2_@BSA nanocomposites had a much higher r_1_ (at 1 T) in acidic conditions (r_1_= 13.08 and 18.76 mM^−1^ s^−1^ at the AMI-mimicking pH 6.5 and the macrophage intracellular pH 5.0, respectively) than under normal physiological conditions (r_1_= 0.34 mM^−1^ s^−1^ at pH 7.4, after incubation for 1, 4 and 12 h). The higher r_1_ of MnO_2_@BSA at pH 6.5 and 5.0 resulted from the release of Mn^2+^ caused by the low pH, which could bind with albumin to increase r_1_ as a result of the greater molecular mass of Mn^2+^-albumin (longer τ_R_ value) and slowing down of proton mobility. The r_1_ of Gd_2_O_3_@BSA was 10.34 mM^−1^ s^−1^ and pH-independent. The nanocomposites were stable in PBS, as indicated by DLS and arsenazo (III) and calcein colorimetric assays to verify the absence of free Gd^3+^ and Mn^2+^, respectively, in the samples. The nanocomposites showed low cytotoxicity to HUVEC cells and H9C2 cardiomyocytes and quite low induced apoptosis (flow cytometry). MnO_2_@BSA accumulated strongly in the AMI regions in rabbit models, as opposed to Gd_2_O_3_@BSA, as well as demonstrating fast excretion after injection, as shown by the high positive MRI contrast enhancement of AMI in rabbit models, with improved diagnostic ability compared to controls [115].

Finally, MnO_2_/PAA NPs of 4.9 nm size showed good biocompatibility and high r_1_ (29.0 mM^−1^ s^−1^) and a low r_2_/r_1_ = 1.8 (1.5 T), resulting in a strong T_1_ contrast enhancement. In vivo magnetic resonance angiography (MRA) of Sprague-Dawley (SD) rats proved that these NPs had better angiographic performance at low-doses than the commercial GBCA Gadovist^®^ (Gd-DO3A-Butrol) [116].

#### 3.3.2. MnO_2_-Based NPs for MRI-Based Theranostics

MRI imaging and therapy of tumors are hampered by their abnormal blood vessel structure, proliferation and metabolic properties of tumor cells. This causes the tumor microenvironment (TME) to show abnormal properties, such as vascular abnormalities, severe hypoxia, high H_2_O_2_ and glutathione (GSH) concentrations and low pH (4.5–5.0), high lactate levels and glucose deprivation due to the upregulated glycolytic metabolism generating lactic acid, which promote tumor cell growth, tumor progression, metastasis, and drug resistance. The tumor tissue heterogeneity prevents complete eradication by monotherapy, leading to recurrence and development of metastasis. TME-responsive MnO_2_-based nanosystems combining several therapeutic modalities and with tunable structures and morphologies, pH-responsive degradation, and catalytic activity, have high potential to improve the efficacy of cancer treatment. Several strategies to modulate the TME have been proposed, including tumor hypoxia relief, excessive GSH depletion, glucose consumption and moderation of the tumor immunosuppressive microenvironment. MnO_2_-based TME modulation benefits cancer therapies, such as PTT, PDT, radiotherapy (RT), SDT, CT, starvation therapy and immunotherapy [117].

Nanoplatforms including MnO_2_ NPs have recently led to important progress in cancer MRI-based theranostics. They can integrate more than one type of synergistic therapy, minimizing multidrug resistance (MDR) and radioresistance. MnO_2_ works as a responsive MRI CA by responding to the acidic pH and reducing environment of the TME by reacting with H^+^/H_2_O_2_ or glutathione (GSH) and generating Mn^2+^ ions, leading to strong T_1_ relaxation [118,119]. The r_1_ value of MnO_2_ NPs is quite low (<0.1 mM^−1^ s^−1^), resulting from the low access of most of its Mn^4+^ ions (effective magnetic moment μ_eff_ = 5.92 μB atom^−1^, Table 1) to bulk water. However, once exposed to acidic H_2_O_2_ or GSH in the TME, they are reduced to large amounts of Mn^2+^ (μ_eff_ = 3.87 μB atom^−1^) ions, resulting in drastic (>50-fold) r_1_ increases and enhancements in T_1w_ MRI contrast within the tumor. This “OFF/ON” behavior makes MnO_2_-containing NPs the responsive CAs with the highest possibility for clinical translation. The contrast enhancement observed in vivo depends mainly on the r_1_ of the CA and its tissue concentration.

The use of multimodal nanoparticles capable of optimizing the advantages and disadvantages of MRI with those of other modalities is also well known [120]. Simple fabrication procedures of multimodal MnO_2_-based theranostic nanoplatforms have recently been developed, with high specific surface area, controllable size and morphologies and easy surface modification, allowing targeted and controllable drug delivery. Some extensive reviews of this field have been published recently [44,88,117,118,119]. In this section, some representative recent examples of MnO_2_-based theranostic nanoplatforms are described, classified according to the imaging techniques, besides MRI, in which they are active, and their main characteristics are summarized in Table 4.

1. Unimodal systems

The first examples illustrate the use of MnO_2_-based theranostic nanosystems with unimodal MRI combined with one or more therapeutic modalities. A theranostic nanoplatform using highly dispersed 2D PEG5000-MnO_2_ exfoliated nanosheets (NSs), surface-loaded with the chemotherapeutic (ChT) doxorubicin (DOX) drug using electrostatic interactions and Mn–N coordinative bonds (PEG5000-MnO_2_/Dox NS), was designed for pH-responsive MRI and drug delivery. Incorporation of the NSs in the acidic TME led to their breakup and disintegration, pH-responsive controlled release of DOX and reduction of MnO_2_ units to release Mn^2+^ ions. A large increase of r_1_ (at 3 T) of the NSs measured in vitro was observed from the initial value of 0.007 mM^−1^ s^−1^ in water to 3.4 and 4.0 mM ^−1^ s^−1^ after soaking in an acidic buffer at pH 6.0 and 4.6 for 2 h. This led to a highly improved performance as a positive MRI CA, which was confirmed by in vivo 3 T MRI tumor imaging of nude mice with a 4T1 cancer xenograft after i.v. injection of the NSs. A strong T_1_ tumor contrast was observed upon reduction of MnO_2_ NSs efficiently endocytosed into cancer cells and triggered by the endosomal and lysosomal intracellular acidic environment (pH ca. 5.0–5.5). The capability of DOX-loaded MnO_2_ NSs for intracellular drug delivery and their therapeutic efficiency was also evaluated in DOX-resistant MCF-7/ADR cancer cells. No antitumor efficacy studies were performed [121].

A cancer cell-specific, endogenous TME H^+^/H_2_O_2_-activated, MnO_2_-containing nanocomposite (AS1411/Ce6–LPMSNs–MnO_2_) was prepared and characterized. MnO_2_ NPs were grown within the pores of large pore silica nanoparticles (LPMSNs), and the photosensitizer chlorin e6 (Ce6) was covalently linked to LPMSNs. After this, the AS1411 aptamer, with high affinity to the nucleolin overexpressed in the plasma membrane of most cancer cells, was immobilized at the LPMSNs surface. In vitro studies in cultured HeLa cells showed that, upon selective cancer cell internalization provided by the aptamer targeting vector, the biocompatible nanocomposite reacted rapidly with lysosomal H^+^/H_2_O_2_ and produced large amounts of O_2_ in situ, which relieved tumor hypoxia and enhanced PDT resulting from production of singlet oxygen (^1^O_2_) by 660 nm laser-irradiated Ce6. At the same time, MnO_2_ acidic dissolution released free Mn^2+^, with an r_1_ = 9.99 mM^−1^ s^−1^ (pH = 6.5, 3T), acting as a “turn on” T_1_ MRI probe in the cultured HeLa cells in real time during therapy. The NPs showed cytotoxicity to HeLa cells under laser illumination in a concentration-dependent manner. A significant photocytotoxicity with an inhibition rate of about 70% was observed even at a 20 μg mL^−1^ concentration. Thus, these biocompatible NPs showed increased therapeutic properties towards targeted cancer cells and increased T_1_ MRI contrast in vitro and provided a new strategy for PDT under tumor hypoxia [122].

Another MnO_2_-based theranostic nanoprobe (b-P25@MnO_2_) was obtained by coating black commercial P25 TiO_2_ (b-P25) with MnO_2_ for GSH-responsive T_1_ MRI contrast and enhanced photothermal therapy (PTT). Upon i.v. injection of b-P25@MnO_2_ NPs into 4T1 tumor-bearing Balb/c female nude mice, the excess GSH in the TME caused release of Mn^2+^ ions by disintegrating the surface MnO_2_ of the nanoprobe, producing an r_1_ = 30.44 mM^−1^ s^−1^ and causing a very good intratumoral and cellular MRI positive contrast. The nanocomposite also showed a stronger PC capability (30.67%) in comparison with isolated b-P25, in cells and in the tumor model under 808 nm laser irradiation, causing the total disappearance of the 4T1 tumor [123].

Multifunctional core/shell NPs for MRI and enhanced PDT of cancer were prepared by coating poly (lactide-co-glycolide) (PLGA) NPs loaded with hematoporphyrin monomethyl ether (HMME) with MnO_2_ shells (PLGA/HMME@MnO_2_ NPs). Upon endocytic uptake of the NPs by MCF-7 tumor cells, the MnO_2_ shells catalyzed the production of intracellular O_2_ from H_2_O_2_, while GSH degraded MnO_2_ into free Mn^2+^. These produced positive tumor contrast in MRI images (3 T) upon injection of the NPs into the tail vein of tumor mice, which was most noticeable at the abdomen, perhaps due to the accumulation of excreted Mn^2+^ through the liver. Degradation of the outer layer of the NPs led to release of the HMME photosensitizer, which, under irradiation, produced cytotoxic ROS (e.g., ^1^O_2_) that damaged the tumor cells in the presence of O_2_ generated in the hypoxic tumor site. The decrease of GSH levels contributed to further inhibition of the produced ROS consumption and greatly enhanced the PDT efficacy. The therapeutic efficacy of the NPs was evaluated using S180 tumor-bearing mice, showing a large tumor volume decrease upon treatment with the NPs under 532 nm laser irradiation. Therefore, this system can improve tumor MRI positive contrast and PDT therapy [124].

Synthesis of multifunctional hyaluronic acid-MnO_2_ nanoparticles (HA-MnO_2_ NPs) was performed by reaction of sodium permanganate with HA in aqueous solution, where HA simultaneously acted as reducing agent, biocompatible and biodegradable surface-coating material and targeting ligand to bind to the CD44 receptor specifically in glioma cells. MnO_2_ NPs were enclosed in HA, forming 80 nm sphere-like particles (TEM), with a hydrodynamic size of 83 nm and ζ = −30.4 mV (DLS). Their dispersions were colloidally stable in water, PBS, saline, and cell culture medium containing 10% fetal bovine serum due to the HA coating. They responded to the TMN conditions by releasing Mn^2+^ and O_2_ upon reaction with H_2_O_2_ at acidic pH, 6.0 (tumor), and 5.0 (lysosomal), as shown by UV-Vis. The r_1_ and r_2_ values of HA-MnO_2_ NPs increased to 13.93, 9.07 and 73.34, 60.82 mM^−1^ s^−1^, respectively, from 1.59, 4.60 mM^−1^ s^−1^ in neutral PBS (pH 7.4) and 1.65, 24.22 mM^−1^ s^−1^ in acidic PBS (pH 5.0) at 3.0 T, upon exposure to H_2_O_2_ and GSH in acidic conditions, which caused the release of free Mn^2+^ from MnO_2_. The NPs showed low toxicity to human umbilical vein endothelial cells (HUVECs) and C6 glioma cells after 24 h incubation. C6 glioma cells incubated with Cy5-labeled HA-MnO_2_ NPs were taken up in the cytosol, as shown by confocal laser scanning microscopy (CLSM) and flow cytometry. Adult male Wistar rats intracranially injected with 5 × 10^5^ C6 glioma cells into the left striatum were i.v. injected with HA-MnO_2_ NPs, exhibiting positive contrast enhancement of the gliomas in T_1w_ MRI (3 T) up to 3 days after one single dose, being able to detect rat intracranial glioma by MRI for prolonged periods. These NPs also alleviated tumor hypoxia in this rat model, which was explained by the downregulation of VEGF and HIF-1α expression in intracranial glioma by the NPs [125].

The basic properties of some very recent examples of MRI-based MnO_2_ theranostic nanocomposites using PTT [126,127,128], PTT/PDT [129], PDT [130] and combined PTT/ChT (gene therapy) [131] are also summarized in Table 4.

2. Multimodal systems

Several examples illustrating MnO_2_-based theranostic nanosystems using multimodal MRI-based imaging combined with one or more therapeutic modalities are now presented. Most of these combine MRI with NIR fluorescence imaging. The first consists of MnO_2_ NPs stabilized with BSA and encapsulated with nanoscale coordination polymer (NCP) shells, which were modified with PEG. The NCP synthesis was based on high-Z hafnium (Hf) ions and included the cisplatin prodrug c,c,t-(diamminedichlorodisuccinato) PtIV-DSP. The core-shell MnO_2_/BSA@NCP(Pt^IV^-DSP)-PEG(Cy5.5) NPs work as a radiosensitizer due to the capability of Hf to strongly attenuate X-rays, and thus enhance radiotherapy (RT). They simultaneously act as a chemotherapeutic agent due to the release of cisplatin induced by the reduction of PtIV-DSP. The in situ O_2_ resulting from the decomposition of endogenous H_2_O_2_ produced by cancer cells and triggered by the MnO_2_ NPs, greatly helped to overcome the radioresistance associated with hypoxia. Within the acidic TME, MnO^2^ decomposed to release free Mn^2+^ to increase T_1w_ MRI contrast. The r_1_ of the NPs increased from 1.75 mM^−1^ s^−1^ to 9.23 mM^−1^ s^−1^ as pH decreased from 7.4 to 6.5. Upon i.v. injection in 4T1-tumor-bearing Balb/c mice, the NPs showed efficient tumor targeting and fast renal excretion, as supported by MRI and biodistribution results. The therapeutic efficacy of the NPs was validated on the S180 tumor model. An excellent in vivo tumor growth inhibition effect resulted in the presence of the NPs plus X-ray irradiation upon the CT/RT treatment combination. Therefore, the NCP composite biodegradable NPs, with no in vivo toxicity and excellent pH/redox/H_2_O_2_-responsive properties, may be used for cancer theranostics as a result of their responsiveness to different TME parameters [132].

**Figure 5:**
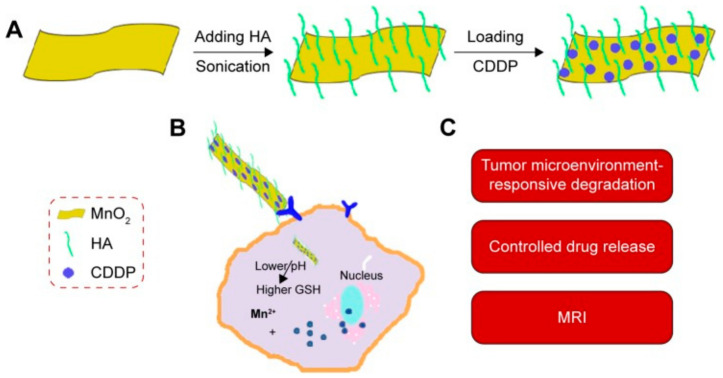
Schematic illustration of nanosheets and their biofunctions. (**A**) Preparation of MnO_2_/HA/CDDP nanosheets; (**B**) the drug release in response to the pH decrease and the GSH increase in the tumor microenvironment; (**C**) the biofunctions of MnO_2_/HA/CDDP nanosheets. Reproduced from Ref. [133].

A redox- and pH-responsive MRI/chemotherapeutic system was designed based on degradable MnO_2_ nanosheets stabilized by functionalization with hyaluronic acid (HA) and with the cisplatin (cis-diamminedichloroplatinum; CDDP) anticancer drug physically absorbed. This MnO_2_/HA/CDDP system efficiently delivered CDDP to tumor cells in vitro and in vivo, triggered by low pH, while the high tumor concentrations of reduced GSH generated free Mn^2+^ ions, with a significant r_1_ increase. Following i.v. injection of MnO_2_/HA/CDDP, anesthetized tumor-bearing mice exhibited positive contrast in the tumor area on T_1w_ MRI (3 T) images. In vivo NIR fluorescence imaging of those mice also showed enhanced tumor fluorescence upon injection with the Cy5.5 dye-labeled MnO_2_/HA/CDDP. The antitumor efficacy of MnO_2_/HA/CDDP was verified in S180 tumor mice, showing that the tumor volume decreased by 20% after four treatments, suggesting a significantly higher effect on slowing tumor progression relative to isolated CDDP administration (Figure 5) [133].

Another example is the pH dual-responsive tumor theranostic platform for MRI and NIR fluorescence detection based on MnO_2_-NSs with a 20–60 nm size, functionalized with 3-aminopropyltrimethoxysilane (APTMS), then with NH2-PEG_2000_-COOH (PEG) and finally conjugated with folic acid (FA) as tumor-targeting group. The DOX chemotherapeutic agent was physically adsorbed onto the NSs. The prepared PEG_2000_-MnO_2_/FA/DOX NSs delivered DOX efficiently to HeLa tumor cells, as shown by confocal microscopy. In the presence of GSH and a slightly acidic pH, the MnO_2_ NSs degraded, releasing free Mn^2+^ ions, causing positive MRI contrast, as shown by in vitro MRI (3 T). The measured r_1_ increased from 0.38 mM^−1^ s^−1^ at pH 7.4 to 2.26 mM^−1^ s^−1^ at pH 5.0 in PBS containing 2 mM GSH. This behavior was confirmed by in vivo MRI (1.5 T) of tumor mice i.v. injected with non-targeted PEG_2000_-MnO_2_/DOX and targeted PEG_2000_-MnO_2_-PEG-FA/DOX NSs, where a positive contrast was observed at the tumor site 4–8 h post-injection, in comparison to normal mice, proving their capability as T_1_ MRI CAs for tumor imaging. In vivo NIR fluorescence imaging of S180 tumor-bearing mice injected with Cy5.5-labeled targeted and non-targeted NSs showed time-dependent accumulation in the tumor through the observed fluorescence intensity, which was strong up to 12 h for the targeted NSs with a maximum at 4 h, while the non-targeted NSs accumulated less at the tumor. The high tumor-targeting ability of the targeted NSs could result from a combined EPR effect and receptor-mediated endocytosis of those NSs. Thus, these NSs, combining MRI with chemotherapy, could be a novel, promising platform for tumor-targeting theranostics [134].

Further examples of recently reported MRI/fluorescence-based TME-responsive cancer theranostic nanoplatforms are: a TME-responsive nanocomposite (Ce6-GA@ MnO_2_-HA-PEG) composed of chlorin e6, gallic acid (GA), MnO_2_, hyaluronic acid (HA), and PEG, for combined PDT/PTT [135]; H-MnO_2_-PEG/Ce6 and DOX for combined PDT/CT/immunotherapy [136]; and a multifunctional nanoplatform consisting of MnO_2_, up-conversion nanoparticles (UCNPs) and the aggregation-induced emission (AIE)-active photosensitizer MeOTTI (PS) (MnO_2_/UCNP/MeOTTI) for combined fluorescence imaging (FLI)-MRI dual-modal imaging-guided PDT [137]. Their properties are summarized in Table 4.

**Figure 6:**
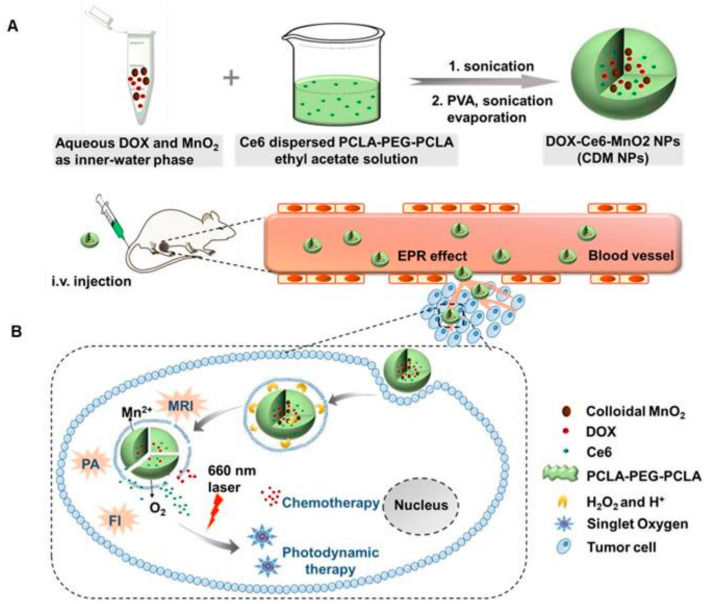
(**A**) Schematic illustration of the hybrid polymeric system consisting of Ce6, DOX, and MnO_2_ co-loaded NPs (CDM NPs) prepared by a double emulsion solvent evaporation method. (**B**) Schematic illustration of tumor-targeting CDM NPs for combined O_2_-generating chemo-photodynamic cancer therapy and trimodal fluorescence (FL), photoacoustic (PA) and magnetic resonance (MRI) imaging. Reproduced from Ref [138].

A MnO_2_-based theranostic nanosystem for trimodal imaging (MRI/NIR-FLI/PAI) and combined therapy (PDT/ChT) was prepared by adding aqueous DOX and colloidal MnO_2_ to a Ce6-dispersed poly (ε-caprolactone-co-lactide)-β-poly (ethylene glycol)-β-poly (ε-caprolactone-co-lactide) (PLCA-PEG-PCLA co-polymer) ethyl acetate solution followed by sonication, to obtain co-loaded DOX-Ce6-MnO_2_@ PLCA-PEG-PCLA NPs (CDM NPs) (Figure 6). The in vivo antitumor effect and MRI/fluorescence imaging properties of the CDM NPs were proven in MCF-7 Balb/c tumor mice and their in vivo PA imaging properties using a multispectral optoacoustic tomography (MSOT) small animal scanner. The mechanism of generation of multimodal contrast is described in Figure 6. Therefore, the CDM NPs could be applied in cancer theranostics by combining chemo-PDT enhanced by O_2_ generation, which was a different way to improve antitumor efficacy [138].

Some MnO_2_-based theranostic nanosystems with bimodal detection combine MRI with X-ray CT imaging. In this example, NPs were designed to overcome the serious limitation of PTT in tumor treatment by the low tissue penetration of lasers used in that therapeutic modality. Alternatively, in this study, radiofrequency (RF) was used for tumor hyperthermia, due to its better tissue penetration. Poly(lactic-co-glycolic acid) (PLGA) NPs were loaded with gold nanorods (AuNRs) suitable for RF hyperthermia and docetaxel (DTX) for chemotherapy. Finally, the NPs were surface coated with ultrathin MnO_2_ to prepare PLGA/AuNR/DTX@MnO_2_ for drug delivery. This nanosystem was tested in vitro (using MCF-7 human breast cancer cells) for combined chemotherapy and RF hyperthermia. Rapid DTX release occurred in the presence of GSH at pH 5.0 due to the combined effect of GSH and acidic pH, which degraded the MnO_2_ coating, causing controlled DTX release. It also produced free Mn^2+^ (solution r_1_ = 1.52 mM^−1^ s^−1^), an efficient T_1_ MRI CA, which, combined with the presence of AuNRs, led to efficient X-ray CT imaging CA, making the nanosystem a bimodal MRI/CT agent. These studies were confirmed in vivo, on subcutaneous S-180 tumor Kunming mice model, where the controlled drug release in the tumor region was promoted by RF hyperthermia and the degradation of MnO_2_ in the TME. Injection of the NPs in the tail vein of the tumor mouse showed clear positive MRI contrast and CT contrast imaging at the tumor site, which was highest at 4 h after administration, as observed on a clinical 3 T MRI scanner and an X-ray scanner. The combination therapy of AuNRs-induced RF hyperthermia and DTX-induced chemotherapy achieved efficient tumor inhibition [139].

Another example of a MnO_2_-based theranostic nanosystem consisted of PVCL-Au-MnO_2_ nanogels using bimodal imaging (MRI/CT) for radiotherapy (RT) combined with chemodynamic therapy (CDT) in tumor hypoxia relief and GSH depletion mechanisms for TME modulation, whose properties are summarized in Table 5 [140].

Finally, some MnO_2_-based theranostic nanosystems with bimodal T_1_/T_2_ MRI detection have been reported. One example consists of DOX-loaded hollow manganese/cobalt oxide nanoparticles (MCO-DOX NPs) with tunable size through a redox reaction and the Kirkendall effect for cancer imaging and drug delivery. The degradation of MCO-70-DOX NPs (70 nm size) by GSH in the tumor TME led to 2.24- and 3.43-fold enhancements of r_1_ and r_2_, respectively, and dual enhancement of the tumor signal in T_1w_/T2_w_ MRI images, as well as the release of the antitumor DOX drug [141].

Many more examples of MnO_2_-based theranostic nanosystem studies using other combinations of imaging and therapeutic technologies are described elsewhere [44,88,117,118,119].

**Table 4:** Overview of fundamental characteristics of some of the examples of MnO_2_-based NPs as MRI (and multimodal/theranostic) CAs covered in this section.

Imaging Modalities	NPs Components	MnO_2_ Reducing Agent in TME	TME Modulation Mode	Therapeutic Modalities	Animal Cancer Model	Reference
T_1_ MRI	PEG_5000-_MnO_2/_Dox nanosheets	H^+^/H_2_O_2_ and/or GSH	Tumor hypoxia relief /GSH depletion	ChT (DOX)	In vivo 4T1 tumor nude mice	[121]
T_1_ MRI	AS1411/Ce6–LPMSNs–MnO_2_	H^+^/H_2_O_2_	Tumor hypoxia relief	PDT	In vitro HeLa cells.	[122]
T_1_ MRI	b-P25@MnO_2_	GSH	GSH depletion	PTT	In vivo 4T1 tumor Balb/C nude mice	[123]
T_1_ MRI	PLGA/HMME@MnO_2_	GSH	GSH depletion	PDT	In vitro MCF-7 cells; in vivo S180 tumor mice	[124]
T_1_ MRI	HA-MnO_2_ NPs	H^+^/H_2_O_2_ and GSH	Tumor hypoxia relief /GSH depletion	down regulation of VEGF and HIF-1α expression	In vitro HUVECs and C6 glioma cells; in vivo C6 glioma tumor Wistar rats	[125]
T_1_ MRI	T-SWITCH: MnO_2_@L-DOPA polymer/sericin	H^+^/H_2_O_2_ and GSH	Tumor hypoxia relief /GSH depletion	PTT	In vitro NIH3T3, HeLa, 4T1 cells; In vivo 4T1-tumor Balb/c mice	[126]
T_1_ MRI	HA-BSA-MnO_2_-PDA (HMDN)	H^+^/H_2_O_2_	Tumor hypoxia relief	PTT	In vitro 4T1 breast cancer cells; in vivo 4T1 tumor Balb/c mice	[127]
T_1_ MRI	Ru/MnO_2_	GSH	GSH depletion	PTT	In vivo H22 tumor Balb/c mice	[128]
T_1_ MRI	MWNT@MnO_2_-PEG@Ce6	GSH	GSH depletion	PDT/PTT	In vitro HeLa cells; in vivo tumor mice	[129]
T_1_ MRI	MnO_2_-polyester-Hyp dendrimer (MHD)	H^+^/H_2_O_2_	Tumor hypoxia relief	PDT	In vitro 4T1 breast cancer cells; In vivo 4T1-tumor Balb/c mice	[130]
T_1_ MRI	MnO_2_/CHA-ICG-DNAzyme@PLGA	H^+^/H_2_O_2_ and GSH	Tumor hypoxia relief /GSH depletion	PTT/ChT(DNA enzyme gene therapy)	In vitro MCF-7 cells; in vivo MCF-7 tumor mice	[131]
T_1_ MRI/OI	MnO_2_/BSA@NCP (Pt^IV^-DSP)-PEG	H^+^/H_2_O_2_ and GSH	Tumor hypoxia relief/ GSH depletion	CT(Pt^IV^-DSP)/RT	In vivo 4T1-tumor Balb/c mice	[132]
T_1_ MRI/NIR-OI	MnO_2_/HA/CDDP and Cy-5.5-labeled MnO_2_/HA/CDDP nanosheets	GSH	GSH depletion	ChT (cisplatin)	In vivo tumor-bearing mice	[133]
T_1_ MRI/NIR-OI	PEG_2000-_MnO_2_/FA/DOX nanosheets	GSH	GSH depletion	ChT (DOX)	In vitro HeLa cells; In vivo HeLa tumor mice	[134]
T_1_ MRI/NIR OI	Ce6-GA@ MnO_2_-HA-PEG	GSH	GSH depletion	PDT/PTT	In vitro HeLa cells; In vivo HeLa tumor mice	[135]
T_1_ MRI/OI	H-MnO_2_-PEG/ Ce6 and DOX	H^+^/H_2_O_2_	Tumor hypoxia relief/immunosuppressive TME moderation	PDT/ChT(DOX)/Immunotherapy	In vitro 4T1 cells; In vivo 4T1 tumor Balb/c mice	[136]
T_1_ MRI/OI	(MnO_2_/UCNP/MeOTTI)	GSH	GSH depletion	PDT	In vivo 4T1-tumor Balb/c mice	[137]
T_1_ MRI/NIR-Fl/PAI	DOX-Ce6-MnO2@ PLCA-PEG-PCLA NPs (CDM NPs)	H^+^/H_2_O_2_	Tumor hypoxia relief	PDT/ChT(DOX)	In vitro MCF-7 cells; In vivo MCF-7 Balb/c tumor mice	[138]
T_1_ MRI/CT	PLGA/AuNR/DTX@MnO_2_	GSH/H^+^	GSH depletion	PTT/ChT(DTX)	In vitro MCF-7 cells; In vivo S-180 tumor Kunming mice	[139]
T_1_ MRI/CT	PVCL-Au-MnO_2_ nanogels	H^+^/H_2_O_2_ and GSH	Tumor hypoxia relief/ GSH depletion	Sensitized RT/CDT	In vitro L929 and Pan02 cells; In vivo Pan02 tumor mice	[140]
T_1_/T_2_ MRI	MCO-DOX NPs	GSH/H^+^	GSH depletion	ChT (DOX)		[141]

### 3.4. MnO_x_ Nanoparticles

The oxidation state of manganese oxide can be determined through characterization techniques like XRD, XPS, XANES, Raman spectroscopy, etc. However, this characterization becomes extremely difficult for nanoscale materials, due to the broadening and weakening of the corresponding peaks and the presence of mixed Mn valence states, including Mn^2+^ (MnO), Mn^3+^ (Mn_2_O_3_), and Mn^4+^ (MnO_2_) in different ratios. Therefore, to avoid inaccuracies, those nanostructures are described simply as MnO_x_ [42].

#### 3.4.1. MnO_x_-Based NPs as MRI CAs

Amorphous MnO_x_/PVP/PAA NPs were synthesized by Rosenholm et al., and their physicochemical properties in the solid state and in aqueous suspensions were compared with the corresponding crystalline MnO/PVP/PAA counterparts. The hydrodynamic properties of these NPs were significantly better than for the corresponding MnO-containing NPs, as was their cytocompatibility for cellular labeling using human mesenchymal stem cells (hMSCs). The relaxivities of the amorphous NPs depended on the reaction times and temperatures of their preparation, e.g., r_1_, r_2_ = 15.6, 30.8 mM^−1^ s^−1^ (MnO_x_, 10 min@120 °C), r1, r2 = 26.7, 52.2 mM^−1^ s^−1^ (MnO_x_, 20 min@120 °C) and r_1_, r_2_ = 24.9, 39.1 mM^−1^ s^−1^ (MnO_x_, 20 min@240 °C) (3 T), which were approximately 10 times higher than those for the crystalline particles. MRI images (3 T) of the amorphous MnO_x_ particles in agar showed differences in the signal intensity profile with the particle concentration. Thus, the NPs provide sufficient MRI contrast to follow cell labeling [142].

In another study, Lin and colleagues synthesized WY-CMC-MnO_x_ NPs by conjugating carboxymethyl chitosan (CMC) with a cartilage-targeting peptide (WYRGRL, termed WY) and then synthesized CMC-assisted MnO_x_ NPs. They showed excellent biocompatibility and a good r_1_ (1.72 mM^−1^ s^−1^ at 7 T). Due to their ultrasmall size (13 nm) and cartilage-targeting ability, these NPs increased the T_1w_ MRI quality of cartilage lesions in vivo upon i.v. injection in osteoarthritis (OA) rat models. The NPs also promoted chondrogenesis in mesenchymal stem cells and enhanced OA therapy through efficient cartilage regeneration after intraarticular injection in destabilization of medial meniscus (DMM) rat models. Thus, the NPs are promising for use in the diagnosis and treatment of early OA due to their detection and repair capabilities [143].

#### 3.4.2. MnO_x_-Based NPs for MRI-Based Tumor Theranostics

Here, we describe some representative recent examples of MnO_x_-based theranostic nanoparticles for tumor diagnosis and therapy, and their main characteristics are summarized in Table 5.

Zhang et al. prepared MnO_x_ NPs integrated into hollow mesoporous carbon nanocapsules (MCNs) as pH-responsive MRI CAs and pH-/HIFU-responsive anticancer drug release. The structure and morphology of the MnO_x_-HMCNs were characterized by TEM, SEM, XPS and Raman spectroscopy and in water dispersions by DLS (hydrodynamic size about 300 nm). The r_1_ value of MnO_x_-HMCNs in neutral buffer solution (0.20 mM^−1^ s^−1^) (3.0 T) increases 2.5-fold to 10.5 mM^−1^ s^−1^ in a pH 4.6 buffer solution, as a result of the breaking up of the MnO_x_ NPs and the Mn^2+^ release in the acidic media. This was observed upon i.v. injection of a MnO_x_-HMCNs solution into nude mice bearing a 4T1 breast cancer xenograft. The tumor margins became much brighter in T_1w_ MRI images (3 T) after 30 min due to the accumulation of the NPs within tumor tissues by the passive targeting EPR effect. The whole tumor was significantly enhanced after 90 min post injection. The carbonaceous framework was also able to include the DOX or camptothecin (CPT) drugs in their large hollow interiors through π-π stacking. Triggered drug release occurred in vitro due to acidic pH and high intensity focused ultrasound (HIFU), as observed by optical microscopy images of DOX and CPT cytotoxicity to MDA-MB-231 breast cancer cells and 4T1 cells, respectively. This report is an important step in the development of new intelligent NPs for cancer therapy [144].

Gao et al. produced technetium-99 (^99m^Tc) surface-labeled MnO_x_-based PEGylated mesoporous silica nanoparticles (^99m^Tc-DTPA-MnO_x_-MSNs-PEG) as bimodal SPECT/MRI CAs to obtain excellent sensitivity and high spatial resolution simultaneously. The NPs formed stable water suspensions with a hydrodynamic diameter of 145.3 nm and ζ potential of −10.8 mV (DLS). The r_1_ of the NPs was 0.63 mM^−1^ s^−1^ in buffer solutions (pH = 7.4) and increased with time at low pH (pH = 5.5), reaching 6.60 mM^−1^ s^−1^ after incubation for 3 h (7 T). This 10-fold increase resulted from free Mn^2+^ ions released from the disintegration of the MnO_x_ NPs. The in vivo pH-responsive properties of the NPs were confirmed by T_1w_ MRI (7.0 T) of tumor-bearing mice, where the tumor showed significant initial (15 min p.i.) contrast enhancement, demonstrating the accumulation of the NPs within the tumor tissue by the passive targeting EPR effect. The radiolabeling yield of the NPs was 99.1% and remained stable. SPECT-CT images of small animals with the MBA-MD-231 breast tumor were acquired at a series of time points after i.v. injection of the NPs. The tumor became clearly visible at 30 min p.i. and reached its highest signal intensity at 3 h p.i., making it easier to distinguish than in the MRI images. The drug loading rate of MnO_x_-MSN to DOX reached a maximum of 382 mg/g, and the drug release was triggered by the degradation of MnO_x_ under mild acid conditions [145].

Dai et al. developed MnO_x_ NPs growing in situ on the surface of tantalum carbide (Ta_4_C_3_) MXene (a family of graphene-analogue 2D materials) nanosheets, which were surface-modified with soybean phospholipids (SP) for trimodal image-guided PTT. The structure of the (MnO_x_/Ta_4_C_3_-SP) NPs was studied by SEM, TEM, HR-TEM, SAED, XRD and X-ray energy dispersive spectroscopy (EDS). The NPs showed in vitro TME pH-responsive and GSH-sensitive T_1w_ MRI capabilities, with a large r_1_ increase due to Mn^2+^ release, which was confirmed by positive contrast of T_1w_ MRI images of the tumor region of 4T1 tumor-bearing mice after i.v. injection of the NPs. The high PC capability of Ta4C3 both in vitro and in vivo endowed the NPs with high photoconductive PTT tumor ablation and in vivo PAI capabilities. Tantalum, with high Z = 73 and high X-ray attenuation coefficient, was used as a high-performance in vivo CT imaging CA in Balb/c mice bearing 4T1 tumors i.v. injected with the NPs. This work offered a new methodological approach addressing both cancer diagnosis and PTT [146].

Chen et al. engineered a new generation of hybrid mesoporous composite nanocapsules (HMCNs) by including small MnO_x_ NPs into hollow mesoporous silica nanoparticles (HMSNs), functionalized with mPEG_5000_ conjugated with FITC and loaded with the DOX drug (MnO_x_@HMCN-mPEG_5000_-FITC/DOX) for Mn^2+^-based pH-responsive dynamic T_1w_ MRI to efficiently respond and detect the acidic TME of HMCNs in vivo in Walker 256 tumor rats. This was integrated with ultrasonographic (US) contrast based on the intrinsic unique hollow nanostructures of HMCNs and observed in vivo in VX2 tumor-bearing rabbits [147].

Ren et al. prepared MnO_x_-coated SPION capped camptothecin (CPT)-loaded MSN (MnO_x_-SPION@MSN@CPT) to control the CPT drug release from the mesoporous silica. MSN NPs (100 nm size) had 2 nm mesoporous channels, which became covered with many nanodots of SPION of 4 nm size coated with an ultrathin layer of MnO_x_. The NPs showed high magnetization (56.1 emu/g) and T_2_ MRI contrast (r_2_ =102.2 mM^−1^ s^−1^, 0.5 T, 310 K) due to the high density of SPION at the surface of MSN, and the MnO_x_ shell degradation in acidic pH typical of the TME provided T^1^ MRI contrast (r_1_ = 13.57 mM^−1^ s^−1^ at pH 5.0) due to the release of Mn^2+^ ions. This was confirmed by in vivo T_2w_ and T_1w_ MRI images of pancreatic tumor-bearing mice after the injection of an MnO_x_-SPION@MSN solution through the tail vein. Thus, this nanoplatform was a TME-responsive T_1_/T_2_ bimodal MRI agent for guided pancreatic cancer chemotherapy both in vitro and in vivo [148].

In the final example, Dong and colleagues synthesized perfluorocarbon PFOB@MnO_x_-PEI-PEG core-shell nanoparticles (PM-CS NPs) to overcome hypoxia-induced ferroptosis resistance. Ferroptosis is a new type of regulated cell death (RCD) based on an iron-dependent oxidative process, in whose initial stage O_2_ is converted into a variety of ROS through the action of enzymes like NADPH oxidase and other redox reactions, which subsequently oxidize the unsaturated membrane lipids through O_2_ addition to their unsaturated bonds, leading to lipid peroxide (LPO) accumulation in the membrane. The depletion of GSH, one of the glutathione peroxidase-4 (GPX4) co-substrates for catalysis of the reduction of LPO, could destroy the redox balance and decrease the expression of GPX4. The critical chemical steps for ferroptosis, ROS production, lipid peroxidation, and GSH depletion, are O_2_-dependent. The hypoxia characteristics of TME reduce the efficacy of ferroptosis-mediated cancer therapy through chemical pathways (ROS production, GSH depletion, and LPO accumulation) and biological pathways (HIF-1α upregulation, and lipid droplet (LD) storage). The PM-CS NPs were characterized by TEM, EDS, DLS (hydrodynamic size = 160 nm, ζ potential = -11 mV, responsible for being colloidally stable in PBS) and UV-Vis. As PFOB is a good O_2_ carrier, PM-CS NPs increase ROS levels, lipid peroxidation and GSH depletion and lower GPX4 activity, compared with MnO_x_ NPs alone. The extra O_2_ relieved tumor hypoxia and broke down the storage of intracellular LDs while increasing the expression of ACSL4 (a symbol for ferroptosis sensitivity). Under the GSH or acidity stimulus, the PM-CS NPs “turned on” the ^19^F-MRI signal of PFOB and activated the T_1_/T_2_-MRI contrast resulting from the release of free Mn^2+^ in vitro. For example, their r_1_ and r_2_ values (1.4 T) increased from 0.41 to 6.3 mM^−1^ s^−1^ and from 5.39 to 43.75 mM^−1^ s^−1^, respectively, upon GSH addition. This was also observed using in vivo ^19^F/^1^H T_1_/T_2_ MRI. PM-CS NPs were intratumorally (i.t.) injected into 4T1 tumor-bearing mice and 1H T_1_/T_2_ MRI and ^19^F MRI images were obtained. The tumor area injected with PM-CS NPs showed a positive contrast in T_1w_ images and a negative contrast in T_2w_ images. The ^19^F MRI image showed a “hot-spot” ^19^F signal in the tumor and a negligible ^19^F signal elsewhere. Thus, PM-CS NPs acted as a pH/GSH-activatable MRI CA for TME-triggered T_1_/T_2_ MRI or ^19^F-MRI of the tumor in vivo. Finally, the PM-CS NPs led to high cancer inhibition for ferroptosis-based therapy by a synergetic combination of O_2_-mediated enhancement of key ferroptosis pathways [149].

**Table 5:** Overview of fundamental characteristics of some of the examples of MnO_x_-based NPs as MRI (and multimodal/theranostic) CAs covered in this section.

Imaging Modalities	NPs Components	MnO_2_ Reducing Agent in TME	TME Modulation Mode	Therapeutic Modalities	Animal Cancer Model	Reference
T_1_ MRI	MnO_x_-HMCNs	H_2_O_2_/H^+^	Tumor hypoxia relief	ChT (DOX, CPT)	In vitro: MDA-MB-231 and 4T1 cells; In vivo: 4T1 tumor mice	[144]
T_1_ MRI/SPECT	^99m^Tc-DTPA-MnO_x_-MSNs-PEG	H_2_O_2_/H^+^	Tumor hypoxia relief	ChT (DOX)	In vitro: MDA-MB-231 cells; In vivo: MBA-MD-231 female BALB/c nude mice and male Kunming mice	[145]
T_1_ MRI/CT/PAI	MnO_x_/Ta_4_C_3_-SP	H_2_O_2_/H^+^ and GSH	Tumor hypoxia relief /GSH depletion	PTT	In vitro: 4T1 cells; In vivo: 4T1 tumor male Balb/c nude mice	[146]
T_1_ MRI/US	MnO_x_@HMSCN-mPEG5000-FITC/DOX	H_2_O_2_/H^+^ and GSH	Tumor hypoxia relief /GSH depletion	ChT (DOX)	In vivo Walker 256 tumor rats (MRI), In vivo VX2 tumor-bearing rabbits (US)	[147]
T_1_/T_2_ MRI	MnO_x_SPION@MSN@CPT	H_2_O_2_/H^+^ and GSH	Tumor hypoxia relief /GSH depletion	ChT (CPT)	In vitro: pancreatic Panc-1 cancer cells; In vivo: pancreatic Panc-1 tumor mice	[148]
^19^F/T_1_/T_2_^1^H MRI	PFOB@MnO_x-_PEI-PEG (PM-CS)	H_2_O_2_/H^+^ and GSH	Tumor hypoxia relief /GSH depletion	Overcome hypoxia-induced ferroptosis resistance	In vitro: 4T1 cells; In vivo: 4T1 tumor mice	[149]

## 4. Conclusions

In this article, recent studies on multifunctional manganese oxide-based nanoparticles as MRI CAs and MRI-based platforms for multimodal and theranostic applications were reviewed. Particular attention was given to their surface modification and functionalization to improve their dispersibility, efficiency as relaxation probes, multifunctionality, targeting capabilities, and minimize their toxicity.

Nanoparticles based on manganese oxides, with the manganese ions in different oxidation states [Mn(II/III/IV)], such as MnO, Mn_3_O_4_, Mn_2_O_3_ and MnO_2_, were extensively explored as alternatives to Gd(III) and high-spin Fe(III) nanosystems in the design of nanoprobes for MRI and theranostics.

Some important points highlighted in this review can be summarized here:

(1) It is known that an ideal T_1_ MRI CA should have an r_2_/r_1_ ratio close to 1, so the morphology and surface passivation properties of the NPs should be optimized, e.g., avoiding non-spherical core geometries, which increase the r_2_ values;
(2) The decreasing number of unpaired electrons in the sequence of Mn^2+^/Mn^3+^/Mn^4+^ ions weaken their paramagnetic character, given by their electronic spin quantum numbers (S = 5/2, 2 and 3/2, respectively), effective magnetic moments and paramagnetic relaxation efficiency. Therefore, the oxidation of Mn^2+^ is, in general, a disadvantage in the development of Mn-CAs;
(3) Due to the low r_1_ relaxivity of MnO_2_-based NPs, they are mainly used for MRI-guided tumor theranostics, where their T_1_ contrast enhancement is provided by the release of free Mn^2+^ in the TME after dissolution of the particle triggered by endogenous stimuli, such as GSH, ROS and acidic pH. Otherwise, excess free Mn^2+^ ions in the body can cause severe toxicity effects and restrict their use to preclinical animal studies;
(4) Besides MnO_2_-based NPs, MnO, Mn_2_O_3_ and Mn_3_O_4_ have also been explored as nano-encapsulated manganese oxide (NEMO) particles, showing their usefulness as T_1_ MRI CAs due to their bright, pH-switchable signal (“OFF” to “ON” at low pH), high metal loading, and targeting capability. Systematic comparison of the different in-house and commercialized NEMOs’ properties, such as synthesis methods, chemistry, release of Mn^2+^ ions, and MRI signal pre- and post-encapsulation within PLGA highlighted their variability [150]. For instance, small MnO NPs produced the highest amount of Mn^2+^ at acidic pH with maximum T_1_ MRI signal, while Mn_3_O_4_ NPs generated the lowest MRI signal. MnO NPs encapsulated within PLGA retained higher Mn^2+^ release and MRI signal compared to PLGA-Mn_3_O_4_ NPs. Therefore, MnO instead of Mn_3_O_4_ should be targeted intracellularly to maximize MRI contrast [151];
(5) A thorough comparison of the imaging effectiveness, biocompatibility, and cost-effectiveness of manganese oxide NPs with Gd^3+^- and Fe^3+^-based NPs as MRI CAs is a task beyond the scope of this review, but should be undertaken. However, the points described above illustrate how the richness of the redox chemistry of manganese (in sharp contrast to gadolinium) can be the basis for their use as responsive NPs, combining T_1_ contrast upon reduction to Mn^2+^ and therapeutic effects associated with ROS generation due to oxidation of Mn^2+^ [23]. Iron oxide NPs, sometimes associated with Mn-NPs, can be very effective as T_1_, T_2_ and T_1_-T_2_ DMCAs and some of them have been previously used clinically [58], while non-oxide iron-based NPs can be efficient T_1_ CAs [25].

Despite the interesting advances highlighted in this review, which make them promising as preclinical MRI contrast agents, manganese oxide-based systems should undertake more in-depth studies, such as in vivo animal experiments investigating their long-term toxicity, biodegradation, elimination mechanisms and biodistribution, for which there is still only limited reported data. Such thorough studies could be used in the future by artificial intelligence techniques to improve their rational design.

## Figures and Tables

**Table 1:** Main chemical and physical characteristics of paramagnetic manganese ions useful for contrast-enhanced MRI applications and comparison with Fe^3+^ and Gd^3+^.

Metal Ion	Free Ion Ground State/Configuration	Spin-Only Magnetic Moment (μ_B_)	Ionic Radius (Å)	Coordination Number	Electronic Relaxation Times (s)	M-H_w_ Distance (Å)
Mn^2+^	^6^S_5/2_ (3d^5^)	5.9	0.83/0.90	6/7 (high spin)	10^−10^–10^−8^	~2.8
Mn^3+^	^5^D_0_ (d^4^)	4.9	0.645	6 (high spin)	10^−11–^10^−9^	2.6–2.8
Mn^4+^	^4^F_3/2_ (d^3^)	3.9	0.39/0.53	4/6	* n.a.	* n.a.
Fe^3+^	^6^S_5/2_ (3d^5^)	5.9	0.64	6–7	10^−11^–10^−9^	2.6–2.7
Gd^3+^	^8^S_7/2_ (4f^7^)	7.9	1.05/1.11	8/9	10^−10^–10^−8^	~3.0

* Not available.

## Abbreviations

AG = alginate; AMI = acute myocardial infarction; APTMS = (3-aminopropyl)trimethoxysilane; APTES = (3-aminopropyl) triethoxysilane; AuNR = gold nanorod; BBB = blood–brain barrier; BSA = bovine serum albumin; CA = contrast agent; CDT = chemodynamic therapy; CF = carbon framework; ChT = chemotherapy; CNP = carbonaceous nanosphere; CPT = camptothecin; CT = X-ray computed tomography; DLS = dynamic light scattering; DMCA = dual-mode contrast agent; DOPA = 3,4-dihydroxy-L-phenylalanine; DOX = doxorubicin; DPDP = dipyridoxyl diphosphate; DSPE =1,2-distearoyl-sn-glycero-3-phosphoethanolamine; DTX = docetaxel; EDS = energy-dispersive X-ray spectroscopy; EPR = enhanced permeability and retention; FI = fluorescence imaging; FTIR = Fourier transform infrared spectroscopy; EDTA = ethylenediamenetraacetate; EDXS-XRF = energy-dispersive X-ray spectroscopy; FA = folic acid; FAR = folic acid receptor; FI = fluorescein isothiocyanate; FLI = fluorescence imaging; FR = folate receptor; GA = gallic acid; GBCA = gadolinium-based contrast agent; GPX4 = glutathione peroxidase 4; GSH = glutathione; GQD = graphene quantum dot; HA = hyaluronic acid; HAADF = high-angle annular dark-field; HER = Herceptin; HIFU = high-intensity focused ultrasound; hMSC = human mesenchymal stem cell; HPMO = hollow porous manganese oxide; HRTEM = high-resolution transmission electron microscopy; HU = Hounsfield unit; HUVEC = human umbilical vein endothelial cell; ICP-MS = inductively coupled plasma mass spectrometry; IO = iso-octane; LLCNP = lipid liquid crystalline NP; LPMSN = large pore silica nanoparticle; LPO = lipid peroxide; MCAO = middle cerebral artery occlusion; MCN = mesoporous carbon nanocapsule; MDR = multidrug resistance; MGRS = magnetic relaxation switch; MOF = metal-organic framework; MON = manganese oxide nanoparticle; MPS = mononuclear phagocyte system; MRA = magnetic resonance angiography; MRI = magnetic resonance imaging; MSN = mesoporous silica nanoparticle; MSOT = multispectral photoacoustic tomography; MTT = 3-(4,5-dimethylthiazol-2-yl)-2,5-diphenyltetrazolium; MTX = methotrexate; NCP = nanoscale coordination polymer; NIR = near infrared; NG = nanogel; NR = nanorod; NOTA = 1,4,7-triazacyclononane-N,N,N-triacetate; NP = nanoparticle; NSF = nephrogenic systemic fibrosis; OI = optical imaging; PAA = polyacrylic acid; PAI = photoacoustic imaging; PBS = phosphate-buffered saline; PC = photothermal conversion; PDA = polydopamine; PDT = photodynamic therapy; PEG = polyethylene glycol; PEI = polyethyleneimine; PET = positron emission tomography; PI = propidium iodide; PL = photoluminescence; PLA = poly(lactic acid); PLGA = poly(lactic-co-glycolic) acid; PS = polystyrene; PSMA = prostate-specific membrane antigen; PSS = polystyrene sulfonate; PTT = photothermal therapy; PTX = paclitaxel; PVP = polyvinylpyrrolidone; PyC3A = N-picolyl-N,N’,N’-trans-1,2-cyclohexenediaminetriacetate; RBITC = rhodamine B isothiocyanate; RCD = regulated cell death; RF = radiofrequency; ROS = reactive oxygen species; RT = radiotherapy; r_1_ = longitudinal relaxivity; r_2_ = transverse relaxivity; SAED = selected area electron diffraction; SDT = sonodynamic therapy; siRNA = small interfering ribonucleic acid; SPECT = single-photon emission computed tomography; SPION = superparamagnetic iron oxide; STEM = scanning transmission electron microscopy; TEM = transmission electron microscopy; Tf = transferrin; TGA = thermogravimetric analysis; TME = tumor microenvironment; T_1w_ = T_1_-weighted; T_2w_ = T_2_- weighted; US = ultrasound; VBA = vinyl benzoic acid; XANES = X-ray absorption near-edge spectroscopy; XPS = X-ray photoelectron spectroscopy; XRD = X-ray diffraction; ZDS = zwitterionic dopamine sulfonate.
